# Genomic analysis of the blood attributed to Louis XVI (1754–1793), king of France

**DOI:** 10.1038/srep04666

**Published:** 2014-04-24

**Authors:** Iñigo Olalde, Federico Sánchez-Quinto, Debayan Datta, Urko M. Marigorta, Charleston W. K. Chiang, Juan Antonio Rodríguez, Marcos Fernández-Callejo, Irene González, Magda Montfort, Laura Matas-Lalueza, Sergi Civit, Donata Luiselli, Philippe Charlier, Davide Pettener, Oscar Ramírez, Arcadi Navarro, Heinz Himmelbauer, Tomàs Marquès-Bonet, Carles Lalueza-Fox

**Affiliations:** 1 Institute of Evolutionary Biology (CSIC-Universitat Pompeu Fabra), Dr. Aiguader 88, 08003 Barcelona, Spain; 2 Centre for Genomic Regulation (CRG) and Universitat Pompeu Fabra (UPF), Dr. Aiguader 88, 08003 Barcelona, Spain; 3 Universitat Pompeu Fabra (UPF), Dr. Aiguader 88, 08003 Barcelona, Spain; 4 Department of Ecology and Evolutionary Biology, University of California, Los Angeles, 612 Charles E.Young Drive South, Los Angeles, California, USA; 5 Departament d'Estadstica, Universitat de Barcelona, 08028 Barcelona, Spain; 6 Department of Biological, Geological and Environmental Sciences, Lab. of Molecular Anthropology, Universit di Bologna, Via Selmi 3, 40126 Bologna, Italy; 7 Laboratory of Medical and Forensic Anthropology, UFR des Sciences de la Sant, 78180 Montigny-le-Bretonneux, Paris, France; 8 Instituci Catalana de Recerca i Estudis Avanats (ICREA), 08010 Barcelona, Spain; 9 Centre de Regulaci Genmica (CRG), Barcelona 08003, Spain; 10 National Institute for Bioinformatics (INB), Barcelona 08003, Spain; 11 CNAG (Centro Nacional de Analisis Genomico), 08028 Barcelona, Spain

## Abstract

A pyrographically decorated gourd, dated to the French Revolution period, has been alleged to contain a handkerchief dipped into the blood of the French king Louis XVI (1754–1793) after his beheading but recent analyses of living males from two Bourbon branches cast doubts on its authenticity. We sequenced the complete genome of the DNA contained in the gourd at low coverage (~2.5×) with coding sequences enriched at a higher ~7.3× coverage. We found that the ancestry of the gourd's genome does not seem compatible with Louis XVI's known ancestry. From a functional perspective, we did not find an excess of alleles contributing to height despite being described as the tallest person in Court. In addition, the eye colour prediction supported brown eyes, while Louis XVI had blue eyes. This is the first draft genome generated from a person who lived in a recent historical period; however, our results suggest that this sample may not correspond to the alleged king.

The analysis of complete individual genomes is now routinely achieved, providing new data to understand the bases for individuality and also to explore the complexity of modern as well as ancient human diversity^1^,^2^,^3^,^4^,^5^,^6^. In the route towards a better characterization of individual genomes and expanding the application of NGS techniques into new disciplines, the analysis of genomes from historical periods is the next logical step.

In 2010, DNA was retrieved from a pyrographically decorated gourd (Fig. 1 and Supplementary Fig. S1) dated to the French Revolution period^7^. According to a text inscribed in the gourd, it contained a handkerchief dipped by a witness called Maximilien Bourdaloue into the blood of the French king Louis XVI (1754–1793) after his beheading in January 21st, 1793 (Fig. 1). The Y-chromosome haplotype, determined with the AmpFlSTR Identifiler PCR amplification Kit is unique in a current European database with over 21,000 individuals, suggesting that any matching with other individuals would likely support a paternal relationship between them. The subsequent analysis of the mummified head^8^ of the king Henri IV (1553–1610)^9^, separated by seven generations from Louis XVI, provided a partial Y-chromosome profile that with the exception of one genetic marker, was concordant with that found in the gourd's blood. Since one allelic difference is not unexpected in pedigrees of several generations, the results still supported that both remains were paternally related^9^.

However, a later analysis of three male living Bourbons from two different family branches revealed identical Y-chromosome haplotypes that were different to those found in the remains of Henri IV and the gourd's individual^10^. To further explore the possibility that the gourd's blood could belong indeed to the king Louis XVI and shed further light on these contradictory results, we have retrieved the complete genome of the human sample contained within the gourd. The objective was to confirm or deny the authenticity of the sample and to try to correlate the genomic data with certain phenotypical traits reported by historical records that might correspond to the king.

## Results

### Contamination estimates and SNP discovery

**Figure 2:**
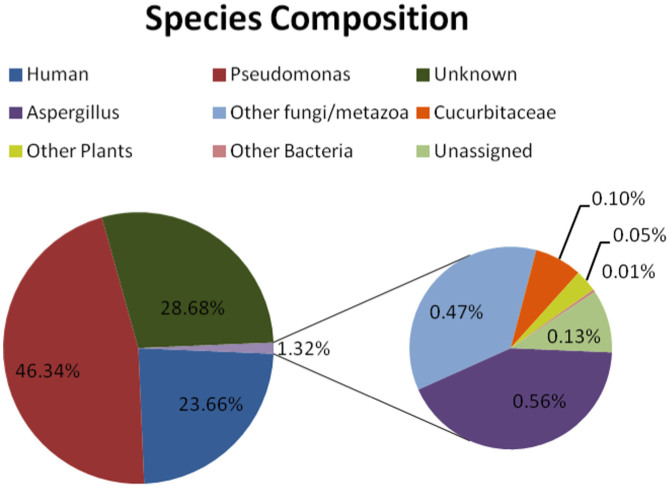
Metagenomics analysis and species composition of the gourd's sample.

We sequenced DNA extracted from the inside of the gourd using Illumina technology (Supplementary Fig. S2 and Supplementary Methods). Due to the inefficiency of the sample, several lanes were used in different platforms resulting in a total 2.5-fold genome wide effective coverage and 7.3-fold exome coverage (Supplementary Tables S1–S2). About 24% of the sequences were human, 46% corresponded to *Pseudomonas* (a group of bacteria comprising plant pathogens), 27% to unknown origin and 1.3% to other organisms, including fungi (0.47%) and of course, Cucurbitaceae (0.1%) (Fig. 2) most likely derived from the gourd itself. The filtered human reads displayed the characteristic DNA damage patterns seen at the end of ancient sequences, although the signal was lower in comparison to that seen in much older samples (Supplementary Fig. S3).

Considering the highly heterogeneous use of a handkerchief and the gourd itself, we anticipated the presence of human contaminants in the sample. To estimate this, we made use of the higher coverage obtained from the mitochondria (~140×, mtDNA genome coverage). Different haplotypes were reconstructed from the mtDNA reads (Supplementary Table S3) with a predominant (73%) N1b1a2 haplotype, concordant with what had been previously determined by polymerase chain reaction (PCR)^7^; however, three additional haplotypes (H1a, J1c2c2 and K*), not shared with people involved in the experimental procedures were present in decreasing ratios (13%, 9.6% and 1.9%, respectively) adding up to a total upper limit of contamination of ~24%.

To obtain nuclear contamination estimates we followed a previously developed procedure^11^ that takes advantage that only one X chromosome is expected to be present in a male individual (and thus, a single allele at each site). The contamination tests are based on the analysis of known polymorphic positions (present at 1000 Genomes Project Phase 1 data^1^) with low coverage (4× to 10×) and the comparison of their mismatch rate to adjacent sites (e.g., less than 5 bases apart). If the sample is contaminated, the number of mismatches at the known polymorphic positions will tend to be significantly higher than in adjacent sites. We obtained a 19.4% (CI 95% = 0.179–0.200) value of potential nuclear DNA contamination (Supplementary Table S4). We also checked the diagnostic haplogroup positions at the Y-chromosome finding a similar value of 17% (CI95% = 0.063–0.277) of contamination (8 out of 47 reads) (Supplementary Table S5). Considering uncertainties associated to sampling and low coverage, it is likely that contamination is equally prevalent at the nuclear and at the mitochondrial genome.

The significant degree of contamination should emerge as heterogeneities in the resulting genotypes. However, we can safely assume that in regions with significant coverage, the 17–24% of background contamination will consistently emerge as the minor allele. Therefore in subsequent analyses we systematically assessed the major alleles.

To remove contaminants by selecting the major allele, we developed a procedure of eliminating SNPs with a substantial skewed allele imbalance (Supplementary Tables S6–S7, Supplementary Fig. S4). Thus, we finally obtained two sets of single-nucleotide polymorphisms (SNPs): a genome wide set of SNPs with >3× coverage (mean coverage = 5×) and a second set of SNPs with >9× coverage (mean coverage = 12×) restricted to the higher coverage exome data. In both sets, we used an allele imbalance removal model accounting for a contamination ratio of up to 30% (Supplementary Methods). While contamination can still remain problematic a lower coverages, we have estimated that in the case of 12× coverage the remaining contamination, according to a Bernoulli statistical model and conditional probabilities, range between 0.25% and 0.39% (Supplementary Methods).

The second set has a higher confidence due to the increased coverage; nevertheless we have calculated a genotype concordance between both datasets of 98%. Obviously, the conservative procedure selected had the side effect of removing real heterozygotes from the gourd's genome. Subsequent Sanger sequencing of 9 sites confirmed the undercalling of heterozygous SNPs (false negative rate of 33.3%) (Supplementary Table S8). Combining the two datasets in a non-redundant set of SNPs, we characterized a total of 1,208,005 SNPs contained in dbSNP, from which 314,549 (26.0%) were reported as heterozygous. Only in the exome, we reported 36,928 SNPs from which 43.75% are heterozygous. The overall Ti/Tv ratio is 1.91 (Supplementary Table S6).

We finally reasoned that our power to make functional interpretations from alleles at individual level could still be compromised; therefore, we applied a final filtering strategy, based on known linkage disequilibrium (LD) patterns in European populations. It is expected that contaminants (we known that there are at least three within the sample) will generate mixed haplotypes, thus breaking the LD blocks on our genome as compared to present-day Europeans. We checked if a random sample of haplotypes found in 60 CEU (Northern and Western European Ancestry) individuals from the 1000 Genomes Project pilot phase^12^ were shared with our genome (Supplementary Methods). We found 17.898 haplotypes (involving ~250,000 SNPs) composed by the same linked variants, ranging in number from 3 to 851 SNPs, and showing an average similarity to the described CEU haplotypes over 93.5% (Supplementary Fig. S5). Moreover, the haplotypes in the gourd's genome tend to be among those found in higher frequencies in modern Europeans (average frequency over 60%) (Supplementary Fig. S6). These observations suggest that despite the contamination of the sample it is still possible to obtain reliable information on the prevalent genome present in the gourd.

### Ancestry inference

According to the historical records, the genealogical track of the king Louis XVI was highly admixed, with a major Central European contribution (Supplementary Table S9). His 16 great-great-grandparents were from present-day Germany (N = 8), Austria (N = 1), Poland (N = 4), Italy/France -House of Savoy- (N = 1), and France (N = 2, one corresponding to Louis, Grand Dauphin of France (1661–1711)) (Supplementary Table S9).

We first determined the gourd's blood paternal ancestry, using diagnostic SNPs in the Y-chromosome, as defined by ISOGG (2014). Several derived SNPs define a G2a2a haplogroup (Supplementary Table S5); again, this result is concordant with that previously found with the AmpFlSTR Identifiler kit^7^.

An identity-by-descent (IBD) tract sharing analysis showed very few IBD tracts with extant Europeans, likely due to gaps in the genomic coverage and undercalling of heterozygous sites. The sequenced individual shared the highest number of fragments with a French individual, but shared the longest tract with a Belgium individual (Supplementary Table S10).

**Figure 3:**
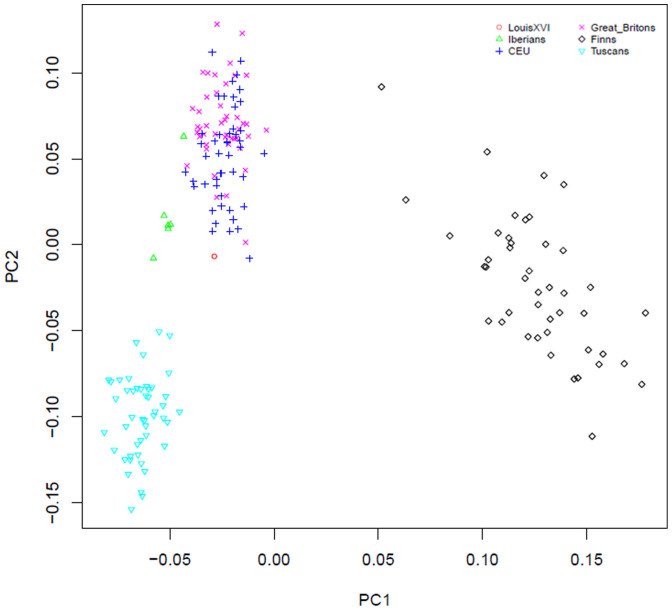
Principal component analysis (PCA) of the gourd's genome SNP data at >9× coverage and compared to European individuals of the 1000 Genomes Project. The analysis was based on 47 randomly selected individuals per population (FINS, CEU, GBR, TSI), except for the Iberians, where all six available individuals were included. A total of 32,701 SNPs were included after filtering for heterozygous positions in order to account for the background contamination.

**Figure 4:**
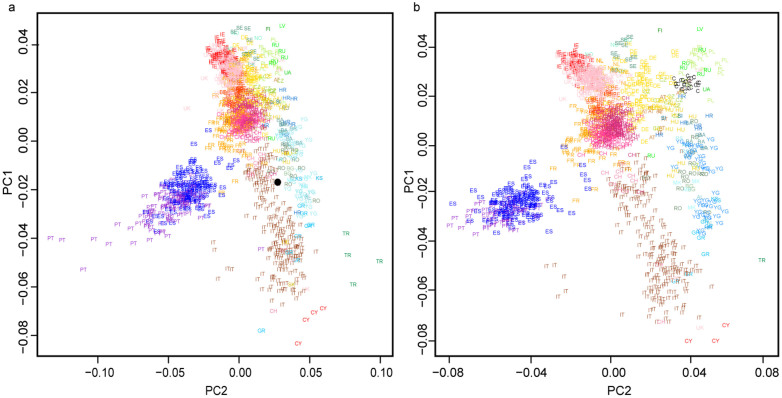
(a) PCA of the merged POPRES dataset and the gourd's genome data. For POPRES individuals, abbreviations and colors for each sample follow conventions of a previously published analysis^24^ in which two letter codes represent population identifiers. The black dot represents the individual data from the gourd's genome. A total of 101,107 SNPs (>3× coverage) were included. (b) PCA of the merged POPRES and 30 randomly composite genomes generated from the known ancestry of Louis XVI at the great-great grandparents generation. For POPRES individuals, abbreviations and colors for each sample follow conventions of a previously published analysis^24^ in which two letter codes represent population identifiers. Composite individuals are represented by uppercase C. A total of 196,526 SNPs were included.

We also performed a principal component analysis (PCA) on the >9× SNP exome dataset, using 236 individuals from the 1000 Genomes Project^1^ for comparison (16,635 SNPs). To minimize differences between our low coverage genome and the reference genotypes, we randomly sampled a single haploid allele from each individual prior to PCA analysis^13^. The gourd's individual can be found close to the CEU distribution, slightly displaced towards the Iberian samples and Tuscan samples from Northern Italy (Fig. 3). Subsequently, to place the gourd's genome in a more precise European context, we performed a genome wide PCA using the European populations of POPRES^14^. Because only 3% of our exome data intersect with POPRES markers, we utilized all SNPs passing our filters genome-wide in this analysis -SNP dataset increased to about 100,000. We found the sequenced individual clusters with Northern Italian individuals (Fig. 4a) which is not what we could expect owing the known ancestry of Louis XVI. This data set is obviously more influenced by the contamination of the sample, due to the less efficient allele imbalance removal associated to the low coverage. To explore if Louis XVI ancestry at the level of his great-great grandparents was compatible with the PCA results, we have randomly generated 30 composite genomes with the proportions of his known ancestry and generated a PCA with POPRES European populations. This simulation showed that the expected position of the king in the PCA is among present day Central European populations (Germany and Poland) (Fig. 4b).

In summary, the results of these analyses do not support the royal identity of the sequenced genome. However, given Louis XVI's complex great-great-grandparental ancestry, the low coverage and the aggressive filtering to remove minor alleles from the current dataset, we consider it could still be possible, although implausible, that the gourd's blood could be that of the French king. Alternatively, it could be that one of the so-called contaminants detected at mitochondrial level may in fact correspond to the king's blood; however, we do not have any evidence (e.g., Bourbon Y-chromosome SNPs among our reads) that could support this assumption.

### Functional exome assessment and phenotypical traits

We performed a functional characterization of the variants found in his genome, including a catalogue of features unique to this individual. A total of 3,850 genetic variants not described in dbSNP were found in the exome (Supplementary Table S11). In the European 1000 Genomes, this figure is on average 16,059 (min = 15,549, max = 16,526); the difference can be attributed to the low coverage and the contamination removal filtering we applied to the gourd's genome. Despite of this, we still found 13 stop codons and 8 non-synonymous changes (all in heterozygosity) within genes that are associated with known human Mendelian diseases, as well as 7 stop codons and 10 non-synonymous changes in genes that are not, but occurred as homozygote for the alternative allele (Supplementary Tables S12–S15). None of these variants could be easily related to any known phenotype -including height- in the king.

We then reviewed different contemporary historical sources, including Marie-Antoinette's correspondence and transcribed conversations from testimonies and relatives (Supplementary Methods) to characterize physical and behavioural traits of Louis XVI. We subsequently contrasted the sequenced genome with several phenotypical features that the king displayed (blue eyes, tall height, obesity), as well as some possible disorders such as type-2 diabetes. The eye colour has an already well established genetic background^15^,^16^, and tools such as IrisPlex^17^ can yield an accurate prediction of eye colour based on the six most-informative single nucleotide polymorphisms (SNPs).

The remaining phenotypes have complex genetic backgrounds that are currently being investigated from genome-wide association studies (GWAS). Due to the gaps and reduction of heterozygotes associated to the low sequence coverage and the background contamination, we don't have information for all the published SNPs; however, we took advantage of linkage disequilibrium (LD) patterns to recapture SNPs not genotyped and also to confirm those present in the genomic draft.

The conservative final set of SNPs after selection and filtering (Table 1) did not allow us to screen for type-2 diabetes and obesity since virtually no data was available to evaluate the risk susceptibility of the individual's genome. Therefore, we continued the analysis on two of the traits, eye color and height.

The king is depicted with blue eyes in all his portraits, despite the fact that his parents (Louis, Dauphin of France, and Maria Josepha of Saxony) were brown-eyed. In the previous study, we retrieved by PCR the critical rs12913832*C SNP at the *HERC2/OCA2* locus, finding the nucleotide substitution associated to blue eyes in heterozygosity. To further explore the gourd's genome phenotype we determined the allelic state at five additional loci and predicted the eye color with Iris-Plex^17^ (Supplementary Table S16). The fact that most of the remaining SNPs are associated with brown eyes resulted in a low probability for blue (2.4%) or intermediate (8.3%) eye color.

Complex traits such as height are more difficult to assess from genetic data alone. However, we could evaluate 23 high-quality SNPs influencing this trait (Table 2). We assumed an additive multiple regression linear model for the increase in centimeters that each allele adds to the final individual's height (based on the Beta-coefficients compiled from literature). To put in context the gain in height inferred for the sequenced individuals, we calculated the distribution of centimeter gain that each combination of height-increasing alleles would confer over an undetermined basal height in Europeans (performing 100,000 resamplings from CEU allele frequencies under Hardy-Weinberg assumption). The allelic combination found in the gourd's genome generates an absolute increase of 7.22 cm. Given that the average increase in the population is 9.05 cm (SD = 1.03), this individual is located in the 3.9 bottom percentile. We repeated the analysis using only the genome-wide significant SNPs (P < 5 × 10^−7^ and <5 × 10^−8^ in GWAS Catalog), but the individual was placed at the 8.37 and 9.78 bottom percentiles, respectively. Similar results were obtained when, rather than assuming HW equilibrium, resamplings were made from the genotypic frequencies in extant CEU samples. In this case, the absolute gain in height for this individual is 5.94 cm, which placed the gourd's individual in the 4.71 bottom percentile (CEU average 6.52 cm; SD = 0.77). The additional search for possible mutations in genes associated to cases of gigantism produced no results.

## Discussion

We have retrieved a draft genome from the gourd's blood, supposed to be that of king Louis XVI, and we have conducted several genomic analyses to characterize this individual. First, we have generated a population analysis with different current European datasets as reference populations. These analyses show that the position of the gourd's individual in the context of modern Europeans is somewhat complex – we see IBD sharing with contemporary French individuals, but also ancestry that is somewhere between contemporary CEU samples and Northern Italian samples. Although it can be argued that Northern Italian populations have clear ancestral ties with Central Europe, this discernible component cannot be easily reconciled with the known Louis XVI family history, given that just one in sixteen great-great-grandparents - Victor Amadeus II, Duque of Savoy (1666–1732)- has some possible Northern Italian ancestry. Additionally, it has to be remembered that the gourd itself has been in Italy for more than a hundred years, but we have no evidence that the contamination found in the sample could be traced back to this period.

Secondly, we have provided a functional interpretation of the gourd's genome, both at the level of disease susceptibilities and also physical traits. Due to the limitations associated to the low coverage, we have focused on the study of height and eye colour.

Contemporary witnesses stated that the king was the tallest person at Court, which was arguably a representation of the best fed people in the country. Some estimates, including one deduced from the length of the coronation cloak in 1775 (measuring 162 cm), placed Louis XVI as an extremely tall person, around 185–190 cm in height or even more^18^. Our estimates suggest the genetic background of the gourd's genome could have predisposed to a height increase of ~7 cm, but this increase is in the lowest range of height increase expected for random present-day individuals of European ancestry. Thus, this individual's height should be only slightly over the average male height in the country that in 1750, as deduced from military French records, was of ~167 centimeters^19^. Despite that some SNPs influencing this trait are likely still unknown, no gene-by-environmental effects have been described so far for height GWAS SNPs. Additionally, height SNPs display a high replicability in other human populations, such as Africans and Asians^20^. Therefore, although it cannot be discarded that the perception of the king's height would be somehow exaggerated by adulation-driven courtesan attitudes, or that other factors (e.g., rare variants not captured by current GWAS) were partially responsible for the king's height, our observation here suggests that the genome is inconsistent with that of a very tall person.

Despite having in heterozygosis a major-effect SNP for blue eye colour, four other SNPs involved in this phenotype display only the allele associated to brown eyes, to the point that the estimated prediction probability is 0.892 for brown eyes. Although this could be considered an upper limit due to the underrepresentation of heterozygous positions in four SNPs not located in the exome (currently between 1–4× of sequence coverage), the prediction for blue eyes could only be increased up to 0.195 if these SNPs were in fact heterozygous. Thus again, within the limit of the quality of our data, the sequenced genome appears to be inconsistent with that of a blue-eyed person.

Finally, we have generated a catalogue of private genetic features, including stop codons and non-synonymous substitutions. If Bourbon genomes contemporaneous to Louis XVI can be retrieved in the future, it would be relevant to the authenticity question to determine if the private mutations found in the gourd's genome are present in members of the French royal family.

Although we cannot totally discard that the gourd's sample belongs to Louis XVI on our genomic data alone, several lines of evidence, including the ancestry analysis and the functional interpretation of the genome fail to provide definitive support for the attribution of this specimen to the beheaded French king. Higher quality genomes of this sample as well as that of other possible genomes from the Bourbon family will be needed to make a more definitive claim.

Second-generation sequencing techniques will allow the generation of massive genomic data in forensic historical cases like those associated to relicts from the French royal family. These data grant more accurate inferences on ancestry and phenotype than those obtained by traditional forensic methods, although some of the difficulties of identifying personal samples remain. With this approach we have obtained and analysed here the first draft genome from a person of a recent historical period. Irrespective of the historical significance of the individual analysed, this kind of genomic data can provide a new source of information on disease susceptibility, demography, mobility patterns and ancestry in historical periods.

## Methods

### DNA extraction, sequencing and mapping

About 60 mg of dried blood sample obtained from the inside of the gourd was extracted in a dedicated ancient DNA laboratory in Barcelona, following experimental procedures described elsewhere^21^.

We generated in several phases a total of 1,545,311,190 reads with Illumina technology (Supplementary Methods). After removing residual primer sequences -a problem expected from the sequencing of short ancient DNA fragments^5^, 214,531,257 reads were mapped to the human reference genome (hg19). PCR duplicates and low quality reads were also removed leaving a final set of 51,507,079 uniquely mapped sequences which correspond to a final genome-wide coverage of ~2.50×. We also used Illumina technologies to sequence the exome (64 Mbps) captured using NimbleGen in-solution capturing technology. A total of 277,762,784 sequences were mapped, from which 9,565,693 remained after removing duplicates, thus achieving a final global coverage of ~7.32×.

### SNP assessment from Linkage disequilibrium patterns

To recapture non-available SNPs and to confirm genotyped SNPs, we first searched for those SNPs present in the NHGRI GWAS Catalog^22^ through a custom-designed pipeline. As a primary input, we intersected the HapMap SNPs (CEU population) with the SNP positions analysed in the gourd's genome. The rationale behind is that any SNP in HapMap would have a frequency high enough to be detected in a GWAS study. This resulted in an input dataset of 2,031,376 SNPs that were further analysed to ascertain LD patterns among them. 100 kbp regions around each of these ~2 million SNPs were defined and all the SNPs with a LD threshold of R^2^ = 1 were considered. We then searched those recaptured SNPs associated to our selected traits at the GWAS Catalog. Afterwards, based on sequence data for the CEU population from the 1,000 Genomes Project^1^, pairwise haplotypes with recaptured and genotyped SNPs were built using PLINK-1.07^23^. With the pairwise haplotypes we could infer if a particular non-genotyped risk allele is segregating in LD with any of the known positions. We subsequently filtered the obtained SNP dataset and considered only those SNPs recaptured by at least three neighboring SNPs, with no incongruities (Supplementary Methods).

Furthermore, the pipeline was used not only to recapture unknown SNPs, but also to verify that the SNPs for which we already had a genotype were correct. We considered as valid those SNPs that were in a congruent haplotype with at least six other SNPs (Supplementary Table S17). Finally, we checked for LD between the recaptured SNPs, to identify putative duplicated tagged regions. We considered that two SNPs constitute a single signal if the LD between them had an R^2^ > 0.8, keeping the one with the lowest *p-value*.

## Author Contributions

D.L. and D.P. collected the sample; A.N., H.H., T.M.-B. and C.L.-F. designed the study; D.L., D.P., contributed to the study design; P.C. performed historical research; I.G., M.M., L.M.-L. and O.R. performed experiments; I.O., F.S.-Q., D.D., U.M.M., C.W.K.C., J.A.R., M.F.-C., S.C., A.N. and H.H. analyzed data; I.O., T.M.-B. and C.L.-F. wrote the paper; all authors discussed the results and commented on the manuscript.

## Additional information

**Accession codes:** The sequencing data have been uploaded to the European Nucleotide Archive under accession code SRX504977 and SRX505016.

## Supplementary Material

Supplementary Information Supplementary Information

## Figures and Tables

**Figure 1:**
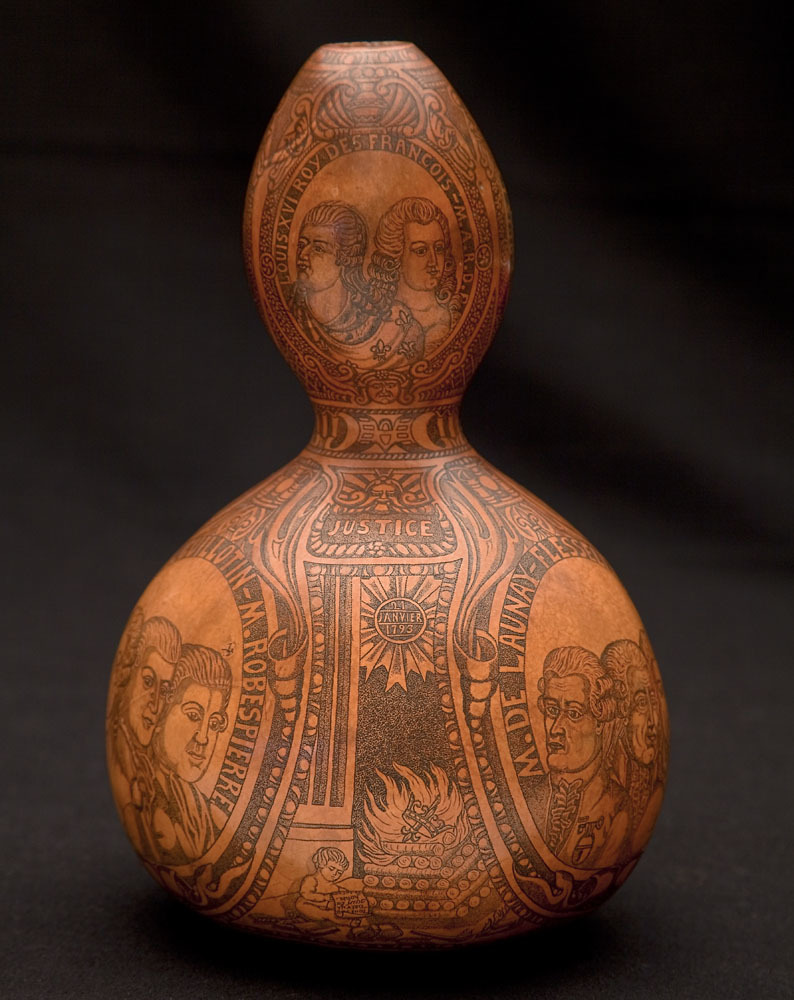
A pyrographically decorated gourd supposed to contain the blood of the king Louis XVI (image and copyright by Davide Pettener).

**Table 1:** Number of SNPs considered for the analysis for each complex phenotype, selected on the grounds of historical accounts about the king Louis XVI

Phenotype	SNPs in GWAS catalog	Recaptured SNPs	After filtering SNPs
Height	357	125	23
Obesity	112	58	--
Type 2 Diabetes	114	59	--

**Table 2:** Height associated SNPs ascertained for the phenotype inference analysis from the gourd's genome. The allelic load column represents the number of height increase alleles for that SNP that our individual carries. EAF (“Effect Allele Frequency”) is the allele that exerts an increase in cm in height. The allelic load can be 0, 1 or 2 depending if the individual carries none, one copy or two copies of the EAF allele, respectively. Mean refers to mean in height of the individuals analyzed and SD is the standard deviation

chr.	region	position	pubmed ID	SNP	EAF	Beta/Allele (cm)	pvalue	mean	SD	gourd's DNA allelic load
12	12p12.2	20758613	18391951	rs11611208	0.067	0.76	2e-06	177.68	6.7	0
3	3q23	141102833	18391951	rs6763931	0.572	0.496	1e-27	177.68	6.7	0
15	15q25.2	84573041	20397748	rs7183263	0.496	0.484	4e-07	178.4	6.92	0
6	6p24.3	7720059	18391951	rs12198986	0.509	0.455	2e-11	177.68	6.7	0
1	1q24.3	172189889	18391951	rs678962	0.885	0.361	3e-08	177.68	6.7	0
6	6q22.32	126835655	18391951	rs1490388	0.571	0.321	6e-07	177.68	6.7	0
6	6p22.3	17699322	18391951	rs12199222	0.257	0.295	7e-07	177.68	6.7	0
12	12p12.2	20857467	20881960	rs10770705	0.367	0.205	8e-18	175.53	6.8	0
12	12p13.2	11855773	20881960	rs2856321	0.397	0.205	5e-15	175.53	6.8	0
4	4q12	57823476	20881960	rs17081935	0.169	0.205	4e-11	175.53	6.8	0
9	9q22.31	95429120	20881960	rs9969804	0.432	0.205	8e-17	175.53	6.8	0
1	1p36.33	2069172	20881960	rs425277	0.239	0.135	2e-08	175.53	6.8	0
4	4p15.31	17944840	18391952	rs16896068	0.881	0.47	2e-13	175.37	6.83	1
3	3q21.3	129050756	20881960	rs6439167	0.212	0.205	9e-15	175.53	6.8	2
15	15q23	70048157	20881960	rs10152591	0.903	0.272	3e-10	175.53	6.8	2
1	1q42.13	227797950	18391952	rs1390401	0.754	0.273	5e-09	175.37	6.83	2
15	15q25.2	84315884	18391951	rs2554380	0.83	0.301	9e-07	177.68	6.7	2
8	8q24.21	130725665	20881960	rs6470764	0.798	0.34	2e-28	175.53	6.8	2
18	18q21.1	46991160	18391952	rs8099594	0.602	0.34	3e-07	175.37	6.83	2
7	7p22.3	2763102	18391951	rs798544	0.699	0.39	7e-15	177.68	6.7	2
2	2q35	219943846	18391952	rs6724465	0.894	0.41	2e-08	175.37	6.83	2
4	4q31.21	145643079	18391952	rs6854783	0.549	0.41	2e-09	175.37	6.83	2
18	18q11.2	20724328	18391951	rs4800148	0.761	0.43	4e-09	177.68	6.7	2
