# Supplementary material for: Genomic analysis of the blood attributed to Louis XVI (1754–1793), king of France
Source: Sci Rep. 2014 Apr 24;4:4666. doi: 10.1038/srep04666 (PMC3998215; doi:10.1038/srep04666)
Supplement: Supplementary Information [file srep04666-s1.pdf]

Genomic analysis of the blood attributed to Louis XVI (1754-1793), king of France

Iñigo Olalde, Federico Sánchez-Quinto, Debayan Datta, Urko M. Marigorta, Charleston W.K. Chiang, Juan Antonio Rodríguez, Marcos Fernández-Callejo, Irene González, Magda Montfort, Laura Matas-Lalueza, Sergi Civit, Donata Luiselli, Philippe Charlier, Davide Pettener, Oscar Ramírez, Arcadi Navarro, Heinz Himmelbauer, Tomàs Marquès-Bonet, Carles Lalueza-Fox

Supplementary Information

Contents

Supplementary Figures 3
Supplementary Tables 9
Supplementary Methods 30
1- The gourd 30
2- Physical and pathological background of Louis XVI 31
3- Sequencing 32
3.1- DNA extraction 32
3.2- Genomic DNA 32
3.3- Preparation of an Illumina sequencing library 33
3.4- Genomic sequencing 33
3.5- Exome selection and sequencing 34
4- Metagenomic analysis 35
5- Genome mapping and coverage estimates 36
6- Contamination estimates 37
6.1- MtDNA affiliation and contamination estimates 37
6.2- X chromosome contamination estimate 38
6.3- Y chromosome affiliation and contamination estimate 38
7- DNA fragmentation pattern 39
8- SNP calling and filtering 40
9- Patterns of post-mortem damage 41
10- PCR genotyping 41
11- Ancestry analysis 42
11.1- Principal component analysis 42
11.2- Analysis of tracts of identity-by-descent 43
12- Functional effect characterization analysis 44
13- Eye color phenotype 44
14- Genetic risk of gourd´s individual 45
14.1- Empirical risk assignment 45
14.2- Risk allele recapturing via pairwise haplotype 46
14.3- Quality control 46
14.4- LD patterns 47
Supplementary References 48

Supplementary Figures

Supplementary Figure S1: the base of the gourd containing the blood attributed to Louis XVI; different text boxes detail the nature and origin of the sample.

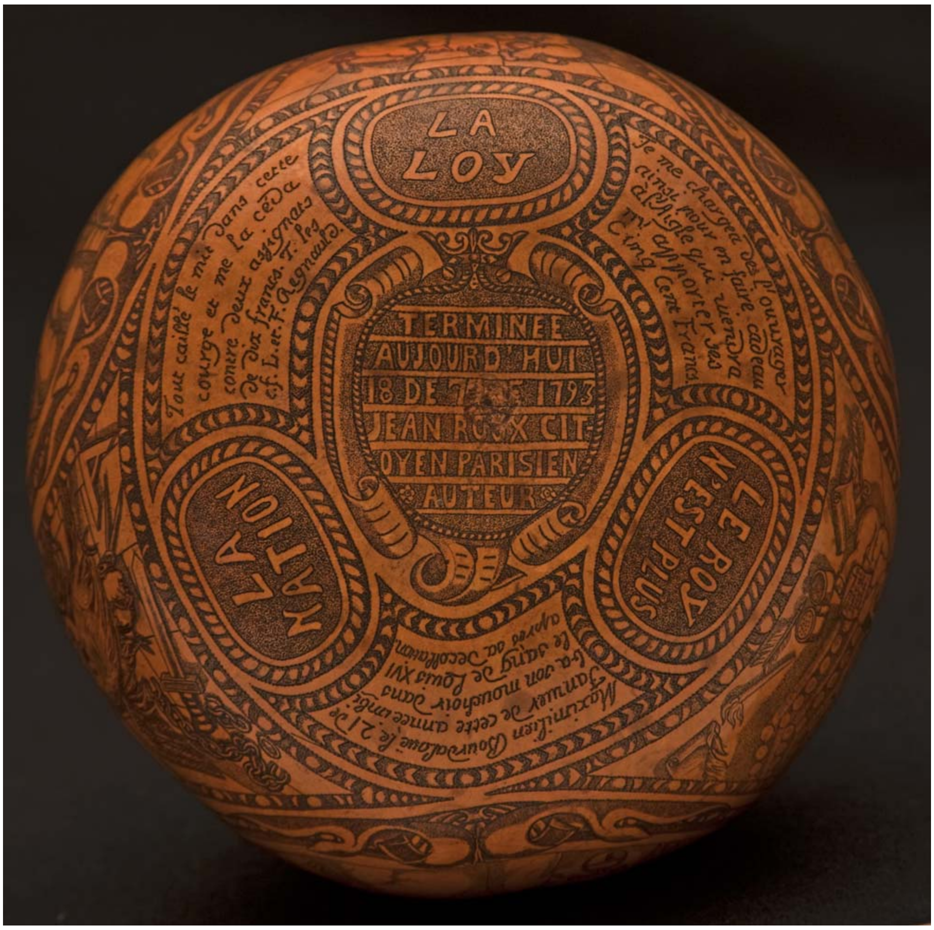

Supplementary Figure S2: Genomic DNA and Illumina libraries. (A) Bioanalyzer analysis of the genomic DNA. (B) Bioanalyzer analysis of the genomic DNA after fragmentation using a Covaris instrument. (C) AgilentBioanalyzer 2100 traces of the obtained Illumina library including 120 bp of Illumina adapters. (D) Insert size distribution plot based on mapping paired-end reads against the human reference genome.

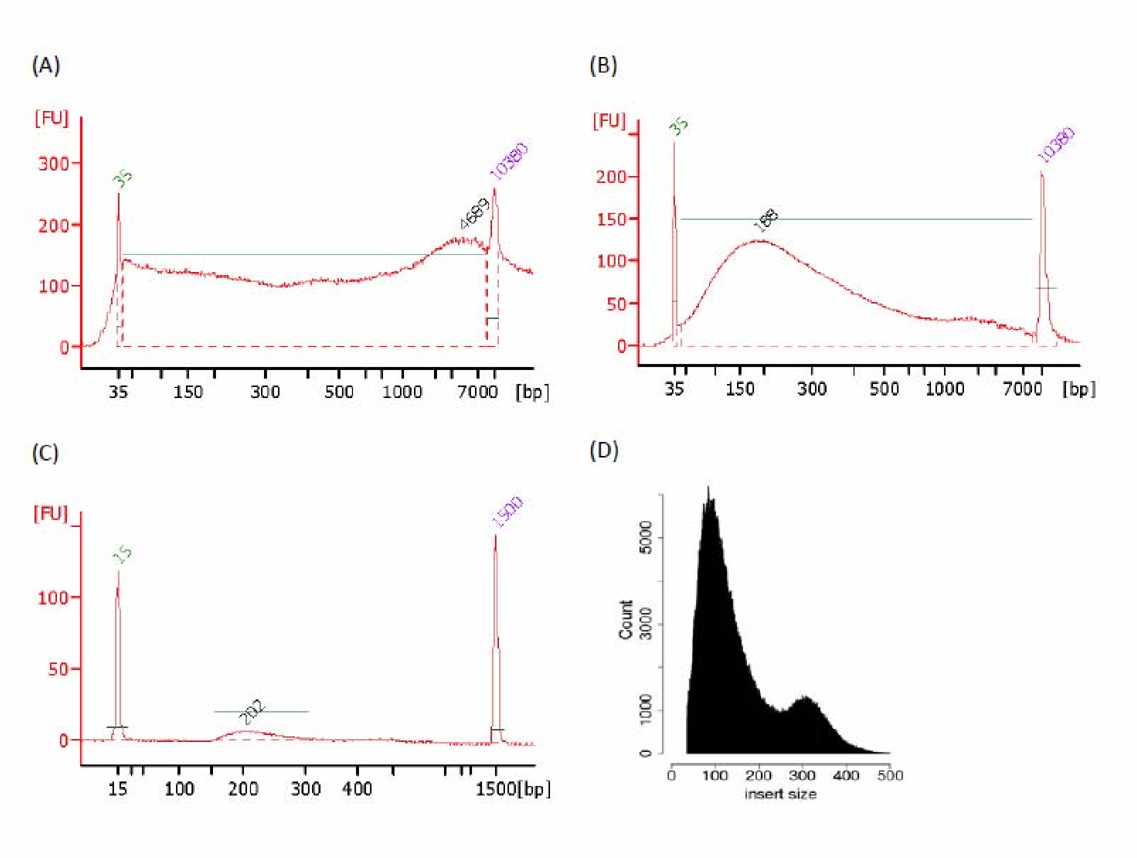

Supplementary Figure S3: DNA fragmentation pattern in the SE exome reads. The base composition of the 10 nucleotides prior and after the ends of the reads is depicted. Left: 5' ends, Right: 3' ends. It can be observed a characteristic ancient DNA bias towards purines prior to the 5' ends of the reads and towards pyrimidines after the 3' ends of the reads.

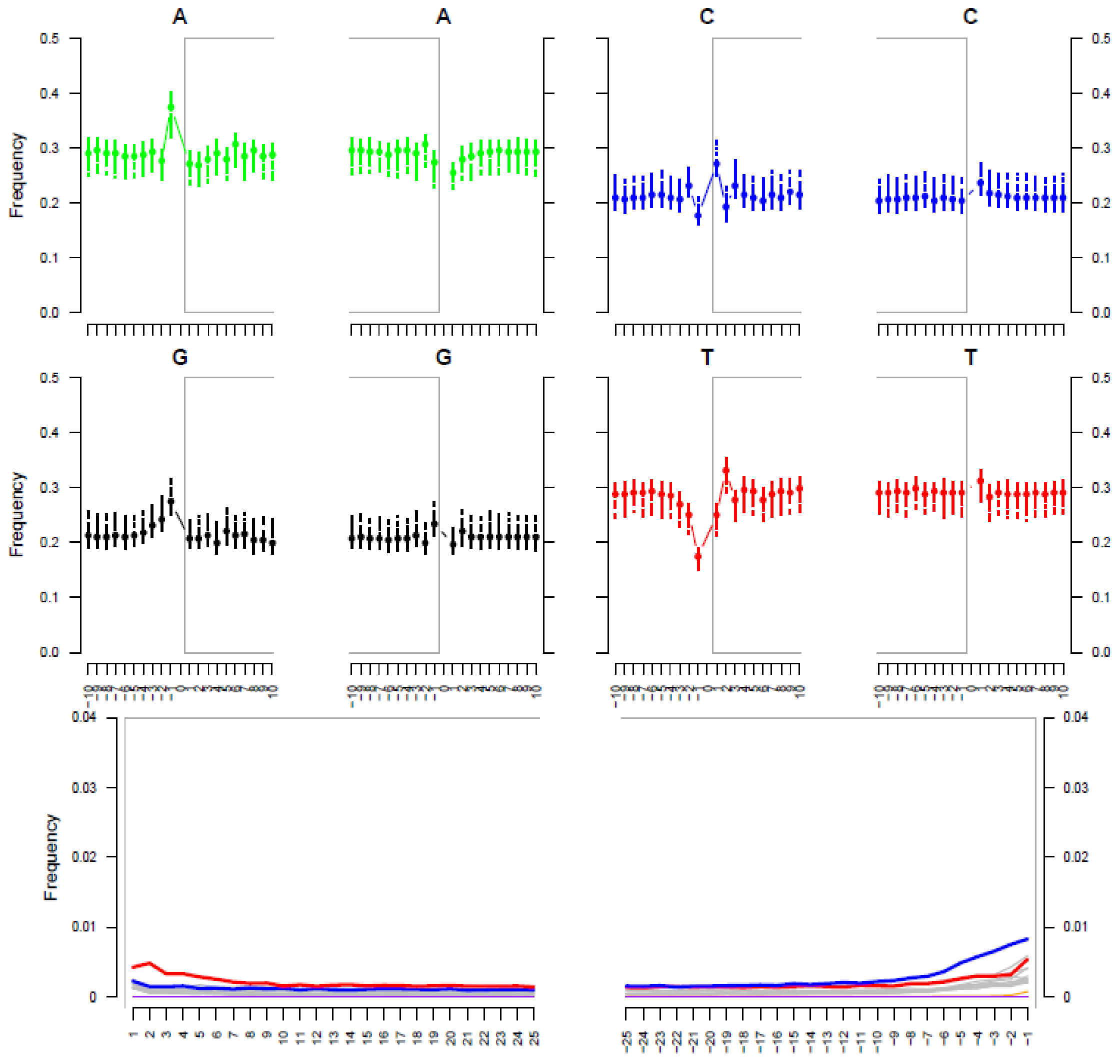

Supplementary Figure S4: Expected decrease in real heterozygous positions as a function on the coverage. Mean coverage for the dataset ≥3x, exome ≥6x and exome ≥9x is 5, 9 and 12 respectively.

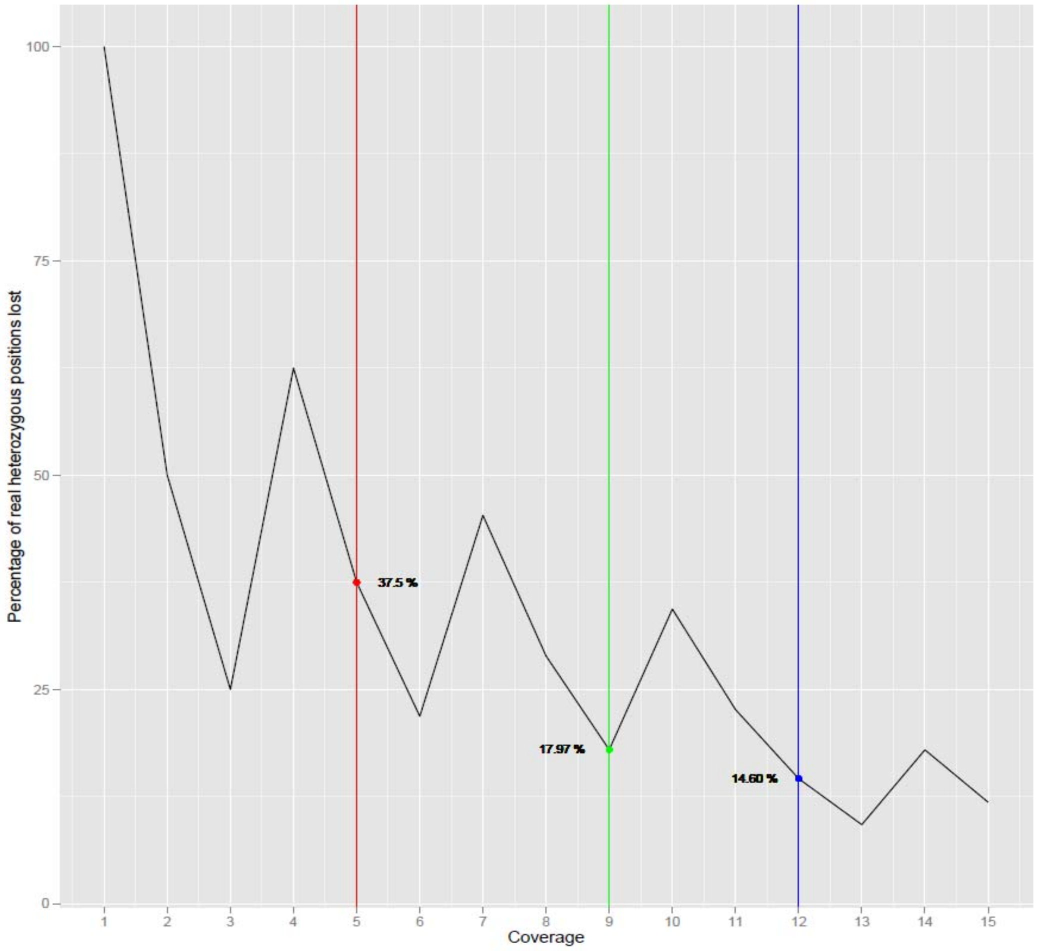

Supplementary Figure S5: Boxplot diagrams representing the distribution of similarity (scale 0 - 1) between gourd's haplotype and CEU haplotype for each of the haplotype length bins.

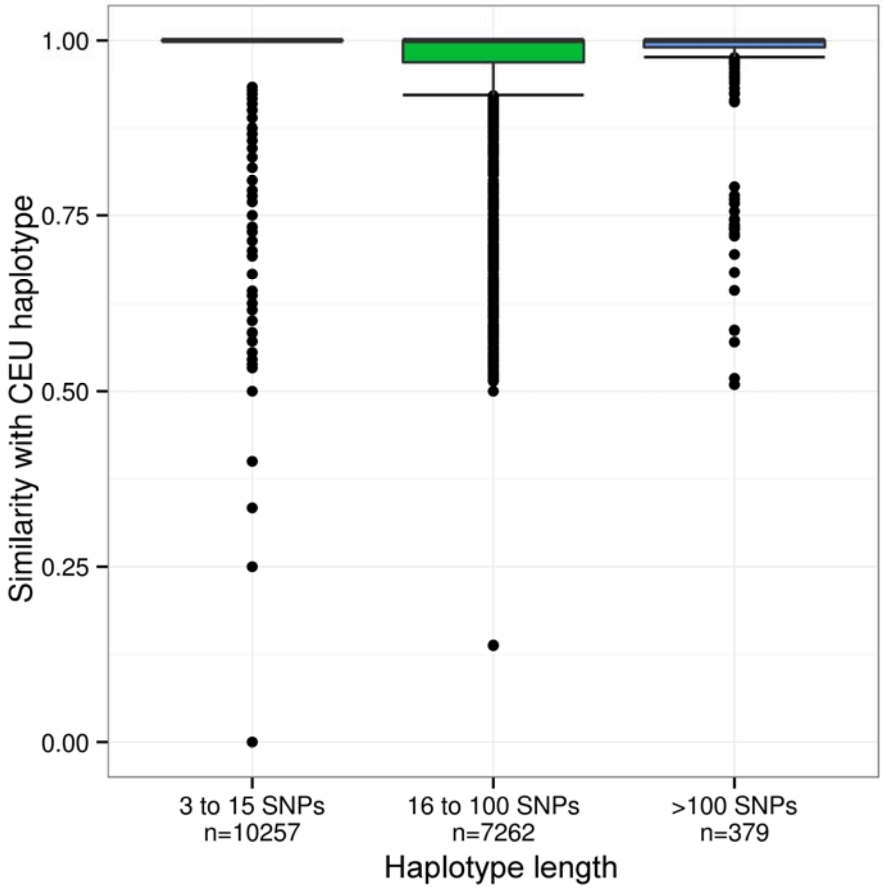

Supplementary Figure S6. Similarity between gourd's haplotype and the most frequent haplotype in CEU and YRI.

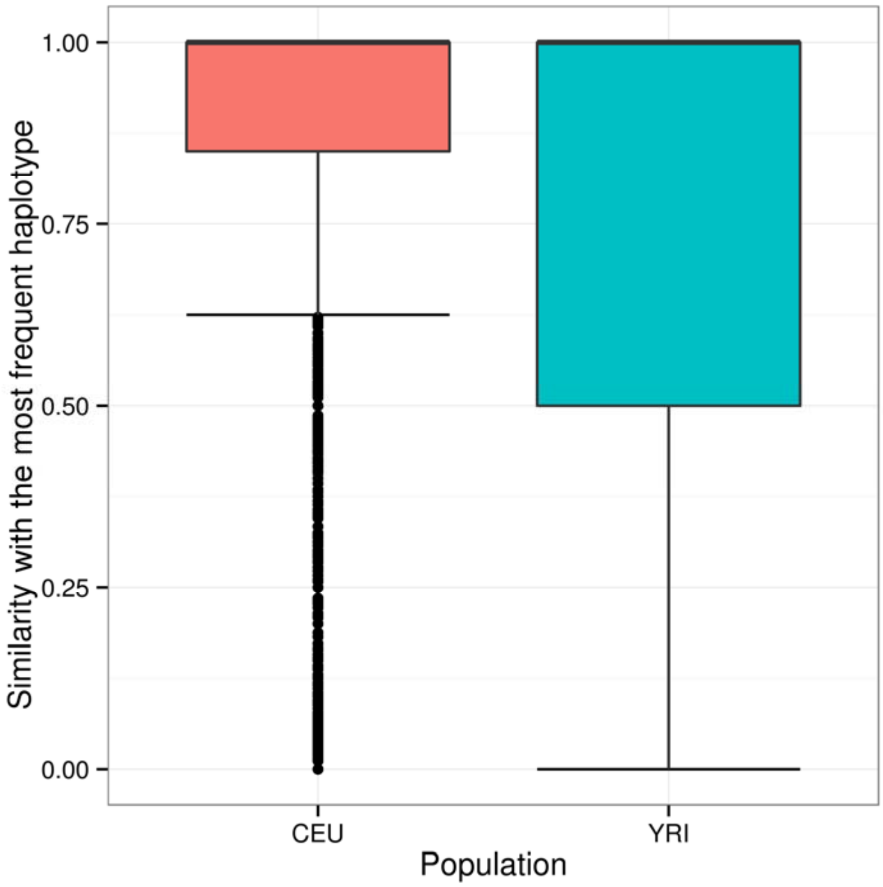

Supplementary Tables

Supplementary Table S1: Sequencing reads after applying AdapterRemoval. Abbreviations: WG = whole-genome; EX = exome-sequencing; PE = paired-end; SE = single-end.

Reactions	# Total reads	# Reads (after adapter removal)	# PE reads	#PE Nucleotides	# SE reads	#SE Nucleotides
WG SE MySeq	6,037,260	5,947,467	–	–	5,947,467	295,803,835
EX SE Gi II	30,582,041	30,310,582	–	–	30,310,582	1,200,587,624
WG	1,545,311,190	874,215,096	223,174,546	21,392,213,006	651,040,550	60,624,941,474
EX	337,912,402	283,733,581	230,308,462	11,472,768,681	53,425,119	3,692,573,586

Supplementary Table S2: Mapping statistics for the different sources of aligned reads. (a) All reads **(b)** Uniquely mapped reads. The mean coverage in EX is referred to the size of exons. The whole genome coverage is estimated from the callable NCBI37 (hg19) regions. Abbreviations: WG = whole-genome; EX = exome-sequencing; PE = paired-end; SE = single-end.

a

**Sample**	**Mapped reads**				**Bases Aligned**	**Mismatch rate**	**Indel rate**	**Strand balance**	**Mean Coverage**
	Total	% Alignment	*After Removing Duplicates*	*Mapqual > 0*					
WG SE MySeq	1,360,737	22.9	1,324,163	1,165,983	56,100,381	0.002	0.0001	0.499	0.024
EX SE Gi II	26,742,574	88.2	2,835,177	1,962,608	75,773,322	0.002	0.0001	0.496	1.18
WG PE	96,152,892	43.1	31,796,378	29,922,638	2,718,380,223	0.003	0.0002	0.5	1.32
WG SE	117,017,628	18	27,399,502	23,644,254	2,498,587,507	0.002	0.0001	0.5	1.22
EX PE	209,828,050	91.1	8,259,577	7,162,700	324,274,841	0.004	0.0002	0.499	5.05
EX SE	41,192,160	77.1	2,286,928	1,757,981	105,128,849	0.004	0.0002	0.496	1.64

b

**Sample**	**Uniquely mapped reads**	**Bases Aligned**	**Mismatch rate**	**Indel rate**	**Strand balance**	**Mean Coverage**
WG SE MySeq	1,096,084	54,203,666	0.002	0.0001	0.499	0.026
EX SE Gi II	1,806,916	71,066,389	0.002	0.0001	0.496	1.11
WG PE	27,414,536	2,602,177,626	0.002	0.0001	0.5	1.27
WG SE	22,996,459	2,469,492,450	0.002	0.0001	0.5	1.2
EX PE	6,117,173	297,518,683	0.003	0.0001	0.499	4.63
EX SE	1,641,604	101,048,699	0.003	0.0002	0.496	1.57

Supplementary Table S3: MtDNA contamination estimate. Coding region mutations and control region mutations defining N1b1a2 haplotype

Nucleotide position	rCRS	LouisXVI	Number of reads	Haplogroup	N1b1a2/total	Percentage of N1b1a2
1719	G	A	130	N1'5	84/130	65%
10238	T	C	128	N1	95/128	74%
12501	G	A	167	N1	132/167	79%
152	T	C	62	N1b	42/62	68%
1598	G	A	106	N1b	73/106	69%
2639	C	T	215	N1b	166/215	77%
5471	G	A	166	N1b	120/166	72%
8251	G	A	78	N1b	59/78	76%
8836	A	G	62	N1b	49/62	79%
16176	C	G	124	N1b	91/124	73%
16390	G	A	145	N1b	104/145	72%
1703	C	T	131	N1b1	106/131	69%
3921	C	A	119	N1b1	90/119	66%
4960	C	T	145	N1b1	95/145	83%
8472	C	T	146	N1b1	110/146	76%
12822	A	G	141	N1b1	102/141	72%
16145	G	A	93	N1b1	75/93	81%
9335	C	T	142	N1b1a	96/142	68%
11362	A	G	133	N1b1a	90/133	68%
4904	C	T	118	N1b1a2	83/118	70%
				Total	1862/2551	73 %

Supplementary Table S4: Results of the tests and contamination rate estimates of the X chromosome contamination analysis. The *p*-values and odd ratios are calculated from a Fisher´s exact test on the contingency tables. Contamination rates are estimated using maximum likelihood with jackknife standard errors. *e* and *e'* denote the number of minor reads at SNP sites and adjacent sites respectively. *d* and *d'* denote the total number of reads at SNP sites and adjacent sites respectively.

	e*/*d	***e'*/*d'***	***p*-value**	**OR**	**ML estimate**	**Standard error (jackknife)**
**Test1**	1564/24218	261/183186	$< 10^{-16}$	45	0.194	0.00531
**Test2**	338/5376	51/40432	$< 10^{-16}$	50	0.189	0.00997

Supplementary Table S5: Y chromosome affiliation and contamination. Phylogenetically relevant positions were ascertained in the gourd´s genome.

SNP name	Position in hg19	Haplogroup	Ancestral	Derived	Number of ancestral reads in the gourd´s genome	Number of derived reads in the gourd´s genome	Total number of reads in the gourd´s genome
L769	23059496	G	G	A	0	1	1
L116	14989721	G	C	G	0	1	1
L154	8614138	G	T	G	0	1	1
L204	5358991	G	C	G	No data	No data	No data
L240	3131153	G	G	T	No data	No data	No data
L269	14958218	G	T	C	No data	No data	No data
L402	15204708	G	T	G	No data	No data	No data
L519	8240725	G	C	T	1	2	3
L520	8700380	G	C	T	No data	No data	No data
L521	9448354	G	A	G	1	5	6
L522	17533325	G	A	C	0	1	1
L523	18957208	G	C	T	0	3	3
L605	18393536	G	G	C	1	1	2
L770	2863466	G	A	T	0	1	1
L836	16896148	G	G	A	0	1	1
L837	17853245	G	A	G	0	2	2
L1258	19431434	G	T	A	2	0	2
L1407	22023296	G	A	G	No data	No data	No data
M201	15027529	G	G	T	No data	No data	No data
P257	14432928	G	G	A	No data	No data	No data
PF3137	2846401	G	C	T	No data	No data	No data
PF2952	14577177	G	G	A	0	3	3
PF2956	14993358	G	A	G	0	2	2
PF2958	15086183	G	G	C	0	2	2
PF3134	15275200	G	C	G	No data	No data	No data
U12	14639427	G	A	C	1	0	1
U21	15204710	G	A	C	No data	No data	No data
U23	14423856	G	G	A	No data	No data	No data
L142.2	6753306	G2	G	A	0	1	1
L156	17174741	G2	A	T	0	1	1
P287	22072097	G2	G	T	0	1	1
L149.1	8426380	G2a	T	G	No data	No data	No data
L31	14028148	G2a	C	A	0	1	1
P15	23244026	G2a	C	T	0	1	1
L1259	15615340	G2a2	C	G	0	2	2
PF3146	5688132	G2a2a	C	T	No data	No data	No data
PF3147	7738069	G2a2a	G	A	No data	No data	No data

PF3151	9785736	G2a2a	A	G	No data	No data	No data
PF3159	14815695	G2a2a	C	G	No data	No data	No data
PF3161	15702713	G2a2a	A	C	0	2	2
PF3165	16582411	G2a2a	C	A	0	1	1
PF3166	16735582	G2a2a	T	G	0	1	1
PF3167	16791005	G2a2a	G	C	No data	No data	No data
PF3168	17572142	G2a2a	T	C	0	1	1
PF3172	18129746	G2a2a	A	C	No data	No data	No data
PF3175	18962113	G2a2a	C	T	No data	No data	No data
PF3176	21185138	G2a2a	G	C	No data	No data	No data
PF3180	21600446	G2a2a	A	T	0	1	1
PF3181	21808944	G2a2a	C	A	No data	No data	No data
PF3182	21822756	G2a2a	C	T	No data	No data	No data
PF3184	22576860	G2a2a	C	T	1	0	1
PF3185	22894488	G2a2a	C	T	No data	No data	No data
PF3186	23291704	G2a2a	T	C	1	0	1
					**Total**	39	47
					**Contamination**	17%	

Supplementary Table S6: Results of the SNP analysis. Ti=transition; Tv= transversion

	All reads		Uniquely mapped reads	
	**Genome-wide**	**Exome**	**Genome-wide**	**Exome**
# Processed loci	3,101,788,170	70,903,267	3,101,788,170	70,903,267
# Called loci	1,208,005	36,928	1,188,465	34,018
# Variant loci	1,208,005	36,928	1,188,465	34,018
Variant rate	3.89×10^{-4}	5.21×10^{-4}	3.83×10^{-4}	4.80×10^{-4}
Base pairs per variant	2,567	1,920	2,609	2,084
# Total SNPs	1,208,005	36,928	1,188,465	34,018
# heterozygous	314,549	16,155	310,790	13,226
# homozygous	893,456	20,773	877,675	20,792
# Singletons	314,549	16,155	310,790	13,226
Heterozygosity	1.01×10^{-4}	2.28×10^{-4}	1.00×10^{-4}	1.87×10^{-4}
Base pairs per HET	9,861	4,388	9,980	5,360
HET-to-HOM ratio	0.35	0.78	0.35	0.64
# Ti	785,741	21,728	779,151	20,482
# Tv	421,617	15,181	408,867	13,515
Ti/Tv ratio	1.86	1.43	1.91	1.52

Supplementary Table S7: Nucleotide change frequencies in the gourd´s genome SNP calling

	All reads				Uniquely mapped reads			
	Genome-wide		Exome		Genome-wide		Exome	
Reference-alternative	# Loci	Frequency (%)	# Loci	Frequency (%)	# Loci	Frequency (%)	# Loci	Frequency (%)
A-T	41,752	3.46	757	2.05	40,014	3.37	690	2.03
A-C	52,968	4.38	1,066	2.89	51,738	4.35	1,014	2.98
A-G	200,151	16.57	4,501	12.19	200,982	16.91	4,406	12.95
T-A	41,795	3.46	798	2.16	40,048	3.37	727	2.14
T-C	201,805	16.71	4,439	12.02	201,935	16.99	4,378	12.87
T-G	53,008	4.39	1,025	2.78	51,784	4.36	970	2.85
C-A	61,571	5.10	4,329	11.72	59,162	4.98	3,607	10.60
C-T	191,515	15.85	6,368	17.24	187,638	15.79	5,817	17.10
C-G	54,334	4.50	1,481	4.01	53,397	4.49	1,420	4.17
G-T	61,690	5.11	4,171	11.30	59,328	4.99	3,585	10.54
G-A	192,270	15.92	6,420	17.39	188,596	15.87	5,881	17.29
G-C	54,499	4.51	1,554	4.21	53,396	4.49	1,502	4.42
Other	647	0.05	19	0.05	447	0.04	21	0.06

Supplementary Table S8: Verification of SNPs of functional relevance by Polymerase Chain Reaction. * from Lalueza-Fox et al. (2011)

dbSNP #	Association	Primers (Forward/Reverse)	Amplicon size (bp)	Genotype (PCR)	Next-generation sequencing genotype
rs12913832*	Blue eyes	TGTCTGATCCAAGAGGCGAG<br>GATGATAGCGTGCAGAACTTG	67	A/G<br>(N=8,6)	A?<br>(N=1)
rs9525638	Bone mineral density	AAAGATTACTGAGATTAACGAC<br>AAGTTGTAGAGTGGATTAATC	52	C/T<br>(N=25,4)	C?<br>(N=3)
rs10811661	Diabetes type-2	TGTCAGCAGCTCACCTCCAGC<br>CAGATCAGGAGGGTAATAGAC	64	C/T<br>(N=10,4)	C/T<br>(N=2,1)
rs16891982	Black hair	GAGGAAAACACGGAGTTGATG<br>GAAAGAGGAGTCGAGGTTGG	56	C/G<br>(N=11,11)	C/G<br>(N=2,5)
rs3821396	Bipolar disorder	CTGCAGTCAACCTCTTCCCC<br>CCTTCCACTCAGGCAAAGAC	71	C/T<br>(N=10,2)	C/T<br>(N=1,10)
rs6971091	Familial obesity	GAAGAAACTCCAAGCCTCCCAG<br>CCCTTGGTCATTAGCTGAATGAG	57	A/G<br>(N=3,2)	A?<br>(N=6)
rs7474896	Obesity	GTCTAAAATATACAAAGAATTCTCAG<br>ATCTCTTTACTTCTGCCTGTTTGC	71	T/C<br>(N=5,16)	T?<br>(N=3)
rs6092	Obesity	TAGGATGCAGATGTCTCCAGCC<br>ACAGCAGACCCTTCACCAAAGAC	75	A/G<br>(N=7,1)	A?<br>(N=6)
rs11684454	Obesity	AAGAAGCATAGGCCAGGTGC<br>AATCCTCTCGCCTTGGCCTC	64	A/G<br>(N=3,1)	A?<br>(N=2)

Supplementary Table S9: Louis XVI maternal and paternal ancestry. Empty boxes indicate dubious attribution (above: paternal side; below: maternal side).

Teresa Zaleska	Adam Uriel Czarnkowski	Teresa Konstancja Czarnkowska	Krzysztof Opalinski	Stanisław Jan Jabłonowski	Marianna Kazanowska			Marie-Thérèse d’Autriche	Louis XIV	Henriette Adélaïde de Savoie	Ferdinand-Marie de Bavière	Philippe Ier d’Orléans	Henriette-Anne Stuart	Charles Emmanuel II de Savoie	Marie Jeanne Baptiste de Savoie
Sofia Anne Czarnkowska		Jan Karol Opalinski		Anna Jabłonowska		Rafał Leszczyński		Louis de France		Marie Anne Victoire de Bavière		Anne Marie d’Orléans		Victor-Amédée II de Savoie	
Catherine Opalinska				Stanislas Leszczynski				Louis de France				Marie-Adélaïde de Savoie			
Marie Leszczynska								Louis XV							
Louis de France															

				Philippe Guillaume du Palatinat	Elisabeth Amélie de Hesse-Darmstadt	Ferdinand III de Habsbourg	Marie-Anne d’Autriche	Madeleine de Hohenzollern	Jean-Georges II de Saxe	Sophie de Brunswick-Lunebourg-Georges	Frédéric III de Danemark			Erdmann-Auguste de Brandenbourg-Bayreuth	Sophie de Brandenbourg-Ansbach
Bénédicte-Henriette comtesse palatine de Simmern		Jean-Frédéric duc de Brunswick-Lünebourg		Eléonore Madeleine du Palatinat-Neubourg		Léopold Ier		Jean-Georges III de Saxe		Anne Sophie de Danemark		Sophie Louise de Wurtemberg		Christian II ernest de Brandenbourg-Bayreuth	
Wilhelmine de Brunswick-Lünebourg-Kalenberg				Joseph Ier du Saint-Empire				Auguste II de Pologne				Eberhardine de Brandenbourg-Bayreuth			
Marie-Josèphe d’Autriche								Auguste III de Pologne							
Marie-Josèphe de Saxe															

Supplementary Table S10: Summary of the IBD tract sharing between gourd's individual and another POPRES European individual

POPRES population	Number of shared segments with gourd's individual	Mean length of the shared segments (cM)
France	3	2.84
Ireland	1	2.812
Yugoslavia	1	2.212
Belgium	1	4.055
Spain	1	1.631

Supplementary Table S11: Summary of the functional analysis of gourd´s individual after allele balance filtering and coverage filtering. NOVEL sites refers to positions not shared with dbSNP137.

Total number of SNPs	14,689
Total number of NOVEL SNPs	3,850
all synonymous-coding sites	2,566
all missense SNPs	3,277
all canceledstart SNPs	4
all readthrough SNPs	4
all nonsense SNPs	163
NOVEL missense SNPs	1,459
NOVEL canceled start SNPs	2
NOVEL readthrough SNPs	0
NOVEL nonsense SNPs	148
SNPs in Splice Acceptor	35
SNPs in Splice Donor	25
SNPs in 5'UTR	1,260
SNPs in 3'UTR	2,475
SNPs in UTR-Splicesites	4
SNPs in Introns	7,517
Polyphen-2 Predictions	2,988
Polyphen-2 Probably damaging	875
Polyphen-2 Possibly damaging	390
Polyphen-2 Benign	1,722
SNPs within CpG Islands	260

Supplementary Table S12: Stop codons within genes associated to Mendelian diseases in the gourd´s genome

Gene	Chr	Position	Nt change	Protein position	AA change	Exon	Private	Hom/Het	Description	Mendelian Disease related to gene	OMIM ID
NEB	chr2	152582053	C/A	106	E/*	6/182	YES	Het	Nebulin	Nemaline myopathy 2	161650
TTN	chr2	179438335	G/T	21607	S/*	275/312	YES	Het	Titin	Muscurlar dystrophy, tibial muscular dystrophy	188840
GLRA1	chr5	151304074	C/A	13	E/*	1/9	YES	Het	Glycine receptor, alpha 1	Hyperexplexia, hereditary	138491
LPL	chr8	19819724	C/G	474	S/*	9/10	NO	Het	Lipoprotein lipase	Hyperlipoproteinemia type I	609708
RP1	chr8	55538031	C/A	530	S/*	4/4	YES	Het	Retinitis pigmentosa 1 (autosomal dominant)	Hypertriglyceridemia-familial, retinitis pigmentosa 1	603937
DBH	chr9	136521683	C/A	491	Y/*	10/12	YES	Het	Dopamine beta-hydroxylase	Dopamine Beta-Hydroxylase Deficiency congenital	609312
GATA3	chr10	8106026	C/A	283	Y/*	4/6	NO	Het	GATA binding protein 3	Hypoparathyroidism, sensorineural deafness, and renal disease	131320
RAG1	chr11	36597333	G/T	827	E/*	2/2	YES	Het	Recombination activating gene 1	Omenn syndrome	179615
FBLN5	chr14	92343917	G/A	367	Q/*	10/11	YES	Het	Fibulin 5	Cutis Laxa	604580
SPTB	chr14	65262234	C/A	489	E/*	11/35	YES	Het	Spectrin, beta, erythrocytic	Elliptocytosis, rhesus-unlinked type, spectrin, beta, erythrocytic	182870
CYP1A2	chr15	75042546	C/A	156	S/*	2/7	YES	Het	Cytochrome P450, family 1, subfamily A, polypeptide 2	Defect in Phenacetin metabolism	124060
SLC5A5	chr19	17999257	C/A	548	C/*	13/15	YES	Het	Solute carrier family 5 (sodium iodide symporter), member 5	Thyroid hormonogenesis I	601843

F9	ChrX	138643736	C/T	298	R/*	8/8	NO	Het	Coagulation factor IX	Hemophilia B	30074

Supplementary Table S13: Stop codons ocurring in genes not associated to Mendelian diseases in which gourd´s individual is homozygous alternative

Gene	Chr	Position	Nt change	Protein position	AA change	Exon	Private	Hom/Het	Description
KHDC1	chr6	74019338	G/A	34	Q/*	1/5	NO	Hom Alt	KH homology domain containing 1
MS4A12	chr11	60265002	C/T	71	Q/*	2/7	NO	Hom Alt	Membrane-spanning 4-domains, subfamily A, member 12
CASP12	chr11	104763117	G/A	125	R/*	3/7	NO	Hom Alt	Caspase 12 (gene/pseudogene)
OR4X1	chr11	48286231	T/A	273	Y/*	1/1	NO	Hom Alt	Olfactory receptor, family 4, subfamily X, member 1
PRMT7	chr16	68373764	C/T	274	R/*	9/19	YES	Hom Alt	Protein arginine methyltransferase 7
PSG8	chr19	43268155	C/A	115	E/*	2/5	YES	Hom Alt	Pregnancy specific beta-1-glycoprotein 8
SSX9	chrX	48159160	C/A	125	E/*	6/8	YES	Hom Alt	Synovial sarcoma, X breakpoint 9

Supplementary Table S14: Non-synonymous changes within genes associated to Mendelian diseases in gourd´s genome

Gene	Chr	Position	Nt change	Protein position	AA change	Exon	Grantham Score	GERP score	Private	Hom/Het	Description	Mendelian Disease related to gene	OMIM ID
FLNC	chr7	128485058	G/A	1180	C/Y	21/48	194	5.56	YES	Het	Filamin C, gamma	Filaminopathy	102565
TNFRSF11B	chr8	119945239	G/A	111	R/C	2/5	180	4.33	NO	Het	Tumor necrosis factor receptor superfamily, member 11b	Paget disease, juvenile	602643
FBN1	chr15	48720624	G/A	2306	R/C	57/66	180	5.76	YES	Het	Fibrillin 1	Ectopia lentis,Marfan syndrome,weill-marchesani syndrome	134797
MPO	chr17	56356900	C/A	178	G/W	4/12	184	5.04	YES	Het	Myeloperoxidase	Myeloperoxidase deficiency	606989
KRT13	chr17	39659604	C/A	224	D/Y	3/8	160	4.4	YES	Het	Keratin 13	White sponge nevus of cannon	148065
APP	chr21	27284191	C/A	591	D/Y	14/18	160	4.5	NO	Het	Amyloid beta (A4) precursor protein	Occipital calcifications with hemorrhagic strokes, leukoencephalopathy, arterial dysplasia, cerebral hemorrhage with amyloidosis	104760
SOX10	chr22	38369693	C/A	404	D/Y	5/5	160	5,12	YES	Het	SRY (sex determining region Y)-box 10	Waardenburg-shah syndrome, hypopigmentation syndrome, peripheral demyelinating neuropathy, central dysmyelinating leukodystrophy, and hirschsprung disease	602229
PDHA1	chrX	19377098	G/T	360	D/Y	11/12	160	5,87	YES	Het	Pyruvate dehydrogenase (lipoamide) alpha 1	Leigh syndrome, x-linked pyruvate decarboxylase deficiency	300502

Supplementary Table S15: Non-synonymous changes ocurring in genes not associated to Mendelian diseases in which gourd´s individual is homozygous alternative

Gene	Chr	Position	Nt change	Protein position	AA change	Exon	Grantham Score	GERP score	Private	Hom/Het	Description
C1orf177	chr1	55273580	G/T	126	G/C	4/10	159	4.45	NO	Hom Alt	Chromosome 1 open reading frame 177
FBLIM1	chr1	16096934	C/T	191	S/F	5/6	155	5.24	NO	Hom Alt	Filamin binding LIM protein 1
GORASP1	chr3	39140352	C/A	317	D/Y	8/9	160	4.68	NO	Hom Alt	Golgi reassembly stacking protein 1, 65kDa
SLC9B2	chr4	103964529	A/C	357	F/C	9/13	205	5.93	NO	Hom Alt	Solute carrier family 9, subfamily B (NHA2, cation proton antiporter 2), member 2
CCDC129	chr7	31683410	G/A	809	C/Y	11/14	194	4.07	NO	Hom Alt	Coiled-coil domain containing 129
GRM3	chr7	86416147	C/T	347	R/C	3/6	180	5.02	YES	Hom Alt	Glutamate receptor, metabotropic 3
MCM4	chr8	48878875	C/T	321	R/C	9/17	180	4.61	NO	Hom Alt	Minichromosome maintenance complex component 4
SLC5A12	chr11	26718732	C/A	340	C/F	8/15	205	5.86	NO	Hom Alt	Solute carrier family 5 (sodium/glucose cotransporter), member 12
RHBDF1	chr16	111839	C/A	389	D/Y	8/18	160	4.43	YES	Hom Alt	Rhomboid 5 homolog 1 (Drosophila)
MOV10L1	chr22	50555596	C/T	424	R/C	9/27	180	4.59	NO	Hom Alt	Mov10l1, Moloney leukemia virus 10-like 1, homolog (mouse)

Supplementary Table S16: State of the six IrisPlex SNPs in the gourd´s genome

Chr	Position in hg19	SNP ID	Minor allele	Gourd´s genome
chr15	28365618	rs12913832	T	*
chr15	28230318	rs1800407	A	G (3 reads)
chr14	92773663	rs12896399	G	G (4 reads)
chr5	33951693	rs16891982	C	C (5 reads)<br>G (2 reads)
chr11	89011046	rs1393350	T	C (3 reads)
chr6	396321	rs12203592	T	C (2 reads)

*Heterozygous, retrieved by PCR

Supplementary Table S17: Quality control feature of the SNP pipeline in a real example. For an unknown genotype at one GWAS SNP (rs2236164, in red cells), associated to height, we have this SNP recaptured by seven neighboring SNPs for which the genotype in gourd´s genome is known (green cells). In the columns Haplotype 1 and Haplotype 2 the first allele position corresponds to the gourd SNP and the second position to the SNP at the GWAS. Note that for 6 out of 7 positions the genotype of the king is consistent (homozygote TT for the GWAS SNP), and only in one of the cases (rs736031, blue cell) the genotype of the gourd is discordant. Being a C to T change and having only one read, it is likely that this apparent heterozygous position is in fact a *post-mortem* damage and that the endogenous genotype is CC.

**SNP gourd** *(known genotype)*	**SNP GWAS** *(unknown genotype)*	**Risk Allele**	**Genotype gourd**	**Haplotype 1**	**Hap1F req**	**Haplotype 2**	**Hap2 Freq**
rs224371	rs2236164	C	AA	GC	0.1882	AT	0.8118
rs6060434	rs2236164	C	CC	GC	0.1882	CT	0.8118
rs750487	rs2236164	C	TT	CC	0.1882	TT	0.8118
rs4281980	rs2236164	C	TT	CC	0.1882	TT	0.8118
rs1886696	rs2236164	C	TT	CC	0.1882	TT	0.8118
rs736031	rs2236164	C	TC	CC	0.1882	TT	0.8118
rs224354	rs2236164	C	GG	CC	0.1882	GT	0.8118

Supplementary Table S18: Example of the haplotype comparison between gourd's genome and the extant haplotypes existing in CEU for a track of 16 SNPs in LD with each other of at least R^2 = 0.7. Full similarity can be seen between the gourd's haplotype and the major haplotype in CEU (underlined). This haplotype was selected for analysis. Some very low frequencies haplotypes were omitted.

Haplotypes in CEU	% of similarity with gourd's haplotype	% frequency in CEU
CATCATTACCCTCATT	100	78.33
CATCACTACCCTCATT	93.75	1.66
TCCTATGCGTTCCGCA	18.75	2.5
TCCTGCGCGTTCTGCA	0.0	15.83

Supplementary Table S19. Similarity values for different haplotype lengths.

Haplotype length	Average % of similarity (s.d)	Average frequency in % (s.d)	Pearson's ρ (*p*-value)
3 to 15 (n=10257)	93.57 *(0.126)*	67.92 *(27.4)*	0.35 *(< 0.001)*
16 to 100 (n=7262)	95.04 *(0.109)*	64.87 *(27)*	0.47 *(< 0.001)*
> 100 (n=379)	97.14 *(0.082)*	60.93 *(24)*	0.37 *(< 0.001)*

Supplementary Methods

1- The gourd

The gourd measures 23.7 cm of height, and has a diameter of the base circle of 15.2 cm. It has the typical shape of the species *Cucurbita moschata*. These gourds were used by the fusiliers at the time of the French Revolution to keep gun powder inside. The gourd had been dessicated and all its surface had been richly decorated with a technique known as pyrography (

Supplementary Figure S1).

The names and faces of relevant characters during the French Revolution are displayed in different places along the surface. In some text boxs, the origin and significance of the object is explained. In the above depictions, the faces of Royalists can be seen, including (in French): « Louis XVI roy des François», « Louis le Dauphin», « Necker», « M.(arie) A.(notinette) R.(eine) D.(es) F.(rançois) », « Simon", « Monseigneur» (?), « F.(rère) D.(u) R.(oy) ». In the base depictions, there are characters from the Revolutionary side: « J.Danton, P.Marat, C.Demoulins; S.Mercier, J.Guillotin, M.Robespierre; M.De Launay, Flesselles, Foullon».

In the text boxs it can be read: « Maximilien Bourdaloue le 21 de Januier de cette année imbiba son mouchoir dans le sang de Louis XVI après sa decollation », « Tout caillé le mit dans cette courge et me la ceda contre deux assignats de dix francs. T.[emoignes] les c.[itoyens] f.[rancois] L. et F. Regnauld » and « Terminee / aujourd'hui / 18 de 7[bre?] 1793 / jean roux cit- / oyen parisien / auteur». In another text box it is possible to read: «Je me chargea de l'ouurager ainsi pour en faire cadeau à l'Aigle qui uiendra m'apporter ses Cinq Cent Francs». That is, someone called Maximilien Bourdaloue, a witness of the king's execution, states that he dipped his handkerchief in the blood of the king and put it inside the gourd, entrusting someone called Jean Roux to decorate it, a work he finished in September of 1793. The purpose of the whole thing seems to be the hope of Bourdaloue of selling the object for 500 francs to someone he calls « The Eagle » and that likely refers to the young Napoleon, who just had become a prominent figure at the siege of Toulon. Additionally, there are some masonic and heraldic symbols, as well as the enigmatic sentence «ci gitent les morts qui ne savoient pas vivre» (that is, "here lie the deads that didn't know how to live")

It is not clear how the gourd ended up as a possession of an Italian family in Imola (province of Bologna). It is a family tradition that they owned it since the 19th century, and there is a letter sent to

the Musée Carnavalet in Paris dated to 1901 (where the object is described) that proves a long-term ownership.

2- Physical and pathological background of Louis XVI

According to direct testimonies originating in relatives and witnesses –and also some physicians for retrospective diagnoses[1–14], the French King Louis XVI is described as:

- Very kind;
- Over weighted;
- Tall (1.93 m);
- Violent on animals (thousands killed at hunting, fighting against cats);
- Suddenly angry in case of injustice;
- No adulterine comportment;
- Slightly sunken eyes;
- Bad dental implantation (dental crowding);
- Large and high forehead;
- Probable phimosis (or impotence?);
- Timidity;
- Goodwill;
- Modesty;
- A huge force;
- Austere;
- Serious;
- Reserved;
- Probably myopic (with further use of glasses);
- Blue eyes;
- Blond hairs;
- Intellectual;
- Polyglot;
- High and nasal voice;
- Little and round head;
- Huge appetite;

- Occasional indigestions;
- Big nose without any disproportion;
- Diabetes mellitus type 2 (?);
- Delayed puberty syndrome adipose-genital type;
- Lung tuberculosis (when young at Compiègne);
- Depressive mood (possible bipolar disorder?).

3- Sequencing

3.1- DNA extraction

The entire gourd sample (around 60 mg) was incubated overnight at 56 °C in 10 ml lysis solution (0.5% SDS, 50 mM TRIS, and 1 mg/mL of proteinase K in H_2O). Subsequently the DNA was extracted in three steps of phenol–chloroform/isoamylalcohol, as described elsewhere[15]. The resulting aqueous phase was concentrated to 50 µl using a Centricon-30 filter column (Millipore). A blank control was extracted along the sample. A PCR with nuclear DNA primers was used to check for possible contamination in the extraction reagents; the PCR yielded no amplification bands.

The extraction procedures were undertaken at dedicated ancient DNA facilities at the Institute of Evolutionary Biology (CSIC-Universitat Pompeu Fabra) in Barcelona. The extraction room, inaugurated at the Parc de Recerca Biomèdica de Barcelona (PRBB) in September 2012, has night UV irradiation and positive air pressure and it is located in a different floor from the main laboratory. No previous work with human DNA had been conducted in these facilities.

3.2- Genomic DNA

Genomic DNA was quantified using both NanoDropND-1000 (NanoDrop Technologies) and Qubit dsDNA BR (Invitrogen, Life Technologies) and its integrity was assayed with a DNA High Sensitivity Bioanalyzer chip (Agilent Technologies). High discrepancy between Nanodrop and Qubit measurements were observed, and DNA degradation was detected by Bioanalyzer analysis (**Supplementary Figure S2A**). Consequently, two aliquots of the genomic DNA were prepared for shearing and subsequent purification. Fragmentation was performed on the Covaris S2 instrument (Covaris Inc.), adjusting the settings as follows: 10% duty cycle, intensity 4, and 200 cycles per burst for 90 seconds and 80 seconds, respectively. Then, each sheared sample was purified using a DNA

Clean & Concentration kit (Zymo Research) and eluted in 30ul. Aliquots were quantified by Nanodrop, mixed, and reanalyzed using a DNA High Sensitivity Bioanalyzer chip (**Supplementary Figure S2B**).

3.3- Preparation of an Illumina sequencing library

Fifty-six µl of fragmented DNA was end-repaired for 30 minutes at 20ºC in a final volume of 100µl using a cocktail of T4 DNA polymerase, DNA polymerase (large fragment) and T4 polynucleotide kinase (New England Biolabs). The resultant end-repaired products were purified using the QIAquick PCR purification kit (QIAGEN) and treated with Klenow fragment (3' to 5' exo-) in the presence of 0.2 mM dATP to add a dAMP to the 3' ends of the fragments. The incubation was done for 30 minutes at 37ºC in a final volume of 50µl. The A-tailed products were purified with the MinElute PCR purification kit (QIAGEN) and used in the ligation step in a final volume of 50µl. 1µl of Illumina Truseq adaptors index 6 and 5 µl of T4 DNA ligase (New England Biolabs) were used in the ligation step, for 15 minutes at 20ºC. The ligation reaction was cleaned with the MinElute PCR purification kit and size selection was done using a 2% agarose gel (Low Range Ultra agarose, BioRad). Three fractions were manually excised from the gel, extending from 150bp to 700bp, and purified using the QIAquick gel extraction kit (QIAGEN). Each fraction was recovered in 30µl, and 10µl were PCR amplified with 1.25µl of Illumina PCR cocktail primer and 25 µl of Phusion polymerase mix (New England Biolabs) in a final volume of 50µl. The cycling conditions were: step1, 98ºC for 30 sec; step 2, 98ºC for 10 sec; step 3, 65ºC 30 sec; step 4, 72ºC for 30 sec; step 5, 72ºC for 5 min; with steps 2 to 4 repeated 12 times in total. The amplified samples were purified with the QIAquick PCR purification kit and analyzed with a Bioanalyzer DNA 1000 assay (Agilent Technologies).

3.4- Genomic sequencing

One out the three library fractions had detectable signal on the Bioanalyzer (**Supplementary Figure S2C**). These adapter-ligated fragments were then quantified using the KAPA Library Quantification Kit (KAPA Biosystem). Samples as well as manufacturer-supplied standards were analyzed in triplicate using a 7900HT Fast Real Time PCR System (Applied Biosystems, Life Technologies) and the SDS2.3 software.

Cluster generation was performed using the TruSeq PE Cluster Kit v3-cBot-HS (Illumina) according to manufacturer`s instructions. The library was loaded at a concentration of 10pM into five lanes on a HiSeq2000 instrument. The total number of read pairs (2x100bp PE) was 772,655,595, and

the true insert size of the library was assessed by mapping a subset of read pairs against the human reference genome NCBI37 (hg19) (**Supplementary Figure S2D**). The library was also sequenced on a MiSeq instrument with a final concentration of 8pM. 6,037,260 reads (51 bp SR) were obtained.

3.5- Exome selection and sequencing

Considering the high fraction of environmental contaminant DNA that is usually present in ancient DNA samples, we performed an exome selection to increase the coverage in the coding regions. Fifteen ng from the previously prepared genomic Illumina library (**Supplementary Figure S2C**) were amplified by 15 cycles of PCR [98ºC for 30s, 15 cycles of 98ºC for 10s, 65ºC for 30s, 72ºC for 30s, and a final extension at 72ºC for 5 min] in order to get sufficient material for the exome selection procedure. The following primers were used: Oligo1: 5'-AATGATACGGCGACCACCGAGA-3' and Oligo2: 5'-CAAGCAGAAGACGGCATACGAG-3'.

Exome selection was performed using the NimbleGene SeqCap EZ Human Exome Library v.3.0 (ref. 06465684001, Roche). Briefly, 1 µg of amplified library was hybridized with 4.5 µl SeqCap EZ Human Exome library for 72 hours at 47ºC. 5 µg of COT human DNA (ref.05480647001, Roche) were added to the hybridization reaction in order to prevent non-specific hybridization to highly repetitive DNA elements. After 72 hours, hybridized fragments were bound to streptavidin beads by incubation for 45 minutes at 47ºC. Non-hybridized fragments were removed by several washing steps. Captured DNA was further amplified by 18 cycles of PCR and was analyzed using an Agilent DNA 1000 chip to estimate the quantity and size distribution of the final captured library. qPCR assays of a set of four NimbleGene Sequence Capture (NSC) control loci were used to estimate the fold enrichment in the captured library compared to the initial library (as stated in Chapter 8 of the NimbleGene protocol). All NSC loci showed enrichment, ranging from 27-fold to 1370-fold.

The captured library was quantified by qPCR with the KAPA Library Quantification Kit. Sequencing was performed on both Illumina GAIIx and HiSeq2000. For sequencing on the GAIIx, the library was amplified with Illumina's Cluster Station using the TrueSeq SR Cluster v5 kit (ref. GD-203-5001), and was loaded at a concentration of 8 pM onto the flowcell. Sequencing was performed following a 1 x 36 cycle recipe and using a Trueseq SBS v5 kit (ref. FC-104-5001). 30,582,041 single reads were obtained in one lane. For sequencing on the HiSeq2000, the library was amplified with Illumina's cBOT using the TrueSeq PE Cluster Kit v3 (ref. PE-401-3001, Illumina), and was loaded at a concentration of 9 pM into one lane of the flowcell. 2 x 50 cycles of sequencing were performed using TrueSeq SBS v3 kits (ref. FC-401-3002, Illumina). 168,956,201 read pairs were obtained.

4- Metagenomic analysis

In order to gain insight into species composition of the sequenced sample, a metagenomic analysis was performed. The dataset analyzed was obtained from one lane of HiSeq 2000 sequencing data, consisting of 162,476,152 read pairs. The reads were first mapped to the human genome (NCBI 37, hg19, hgdownload.cse.ucsc.edu/goldenPath/hg19/bigZips/). Mapping was done on trimmed reads (36 bp) using the GEM mapper[16] allowing for 2 mismatches. Based on the mapping statistics for read 1, the non-mapping (NM) reads and their corresponding paired reads were separated out, a total of 125,502,689 read pairs. A preliminary analysis performed on NM reads indicated the presence of a high proportion of reads of bacterial origin matching Pseudomonas, a group of bacteria comprising well-known plant pathogens and soil bacteria. Hence, in a second step, NM reads were mapped to four different Pseudomonas genomes (Pseudomonas fluorescens SBW25 (AM181176.4); Pseudomonas syringae pv. phaseolicola 1448A chromosome (NC_005773.3); Pseudomonas syringae pv. syringae B728a chromosome (NC_007005.1); Pseudomonas syringae pv. tomato str. DC3000 chromosome (NC_004578.1). As before, NM reads and their corresponding paired reads were separated out, and a total of 56,629,186 read pairs remained. To determine the species composition of these remaining reads, they were first quality-filtered and trimmed according to Minoche *et al*[17], i.e. we removed reads if they contained uncalled bases, or if less than 2/3rd of bases in the first half of the read had quality values ≥30. Then, B-tails were trimmed and reads discarded in case that trimming resulted in reads shorter than 25 nt. Read filtering and trimming resulted in 50,857,561 read pairs and 3,178,341 singleton reads. The 50,857,561 read pairs were assembled using Velvet version 1.2.07[18] using a k-mer size of 49, resulting in 197,251 contigs ≥100 bp with an N50 length of 365 bp, and an assembly size of 61,786,230 bp. The contigs were then BLAST searched against the nt database. The BLAST search (version 2.2.25, e-value 1e-10) of the 197,251 contigs to the nt database (ftp://ftp.ncbi.nlm.nih.gov/blast/db/nt.fa, downloaded January 2013) resulted in hits for 59,040 contigs, corresponding to 11,981,994 reads.

The BLAST results were input to MEGAN v 3.9[19], a tool for metagenomic analysis which puts the taxonomic classification of analysed sequences into a phylogenetic context. Of 59,040 contigs with blast hits, 57,603 contigs were assigned a taxonomic classification, while 1,437 remained unassigned. The species tree consisted of two main branches, bacteria and eukaryota. The majority of contigs in the

bacterial branch were assigned to Pseudomonas-group genomes. In the eukaryotic branch there were three main sub-branches – plants, human and fungi. The "Plants" sub-branch consisted mainly of Cucurbitaceae (the family which encompasses gourd), while the "Fungi" sub-branch consisted mainly of Aspergillus. **Figure 2** illustrates the overall composition of the sequenced sample, as a synthesis of short-read mapping and Megan analysis. In summary, the highest proportion of reads with known origin came from Pseudomonas (46%), followed by human (24%) and Aspergillus (0.6%). Cucurbitaceae comprised 0.1% of the data. Considering that plant genomes evolve very rapidly and the genome of gourd is not sequenced, it is conceivable that further reads from gourd are contained within the data but could not be identified.

Exome selection using the same library efficiently removed the environmental DNA background in the sample. After this procedure, 84% of sequencing data could be successfully mapped to the exome human genome.

5- Genome mapping and coverage estimates

Our final genomic dataset was a combination of single-end reads and paired-end reads generated with different strategies (exome vs. whole genome) and machines (Illumina GAII, Illumina Hiseq2000, Illumina MySeq). The use of ancient DNA implies a special treatment to deal with the degradation of the template sequences, which very frequently may be too short and thus produce overlapping sequence reads for a given pair and the sequencing of the adapter; importantly, this may cause an over-representation of certain bases in the reads and thus bias the SNP calling. Consequently, we used AdapterRemoval[20] in order to both remove the adapter sequence from the reads and build a consensus single-end read from those reads in the same pair that covered the same sequence (Supplementary Tables

Supplementary Table S1). In summary, the BAM files for our SNP calling is composed from sequencing efforts from four experiments: (i) whole-genome data single-end (1 run Illumina MySeq), (ii) exome-sequencing single-end (1 run Illumina GAII), (iii) whole-genome sequencing paired-end (5 lanes Illumina Hiseq2000), (iv) exome-sequencing paired-end (1 lane Illumina Hiseq2000) (

Supplementary Table S2).

We used BWA[21] to map all the reads to the human genome (NCBI 37, Hg19) using the default

parameters for single-end and paired-end with the exception of the quality trimming parameter, which was set to a Sanger quality score of 15. On average, 24% of the shotgun and 84% of the exome data was aligned to the human reference genome (NCBI 37).

The final coverage (

Supplementary Table S2) has been estimated from total length of the callable regions (2,052,511,509 nucleotides) extracted from the mapability table of NCBI37, hg19 at UCSC. The coverage on the exome is estimated from the total 64,190,747 nucleotides included in the NimbleGen array used.

6- Contamination estimates

6.1- MtDNA affiliation and contamination estimates

The number of raw reads that aligned to the human mtDNA reference genome (rCRS) was 65,455. After removal of PCR duplicates, we kept 18,324 reads, representing a depth of coverage of 113X. Given that mitochondrial haplogroup of the gourd´s individual was previously classified as N1b based on mutations at the mtDNA HVR1 and HVR2[22], we first checked if the most prevalent haplotype in our data was a N1b haplogroup. All the mutations already described in the control region were found (73G, 151T, 152C, 189G, 194T. 195C, 263G, 315.1C, 16093C, 16145A, 16176G, 16223T and 16390A). Outside the control region we found the following mutations: 750G, 1438G, 1598A, 1703T, 1719A, 1734T, 2639T, 2706G, 3921A, 4769G, 4904T, 4960T, 5471A, 7028T, 8251A, 8472T, 8836G, 8860G, 9335T, 10238C, 11362G, 11719A, 12501A, 12705T, 12822G, 14766T and 15326G. Using HaploGrep[23] and Phylotree version 15 (http://www.phylotree.org[24]) the correspondent haplogroup was assigned. The mitochondrial genome displays all the mutations expected for subhaplogroup N1b1a2, with six additional private mutations: 151T, 189G, 194T, 195C, 1734T and 16093C.

The high coverage obtained for the mtDNA allowed us to search for additional haplogroups present in the sample. We looked for reads not matching N1b1a2 haplogroup in every position that, according to Phylotree version 15, has a mutation defining haplogroup N1b1a2. For the 20 diagnostic positions analyzed, we found 27 % of reads not matching the N1b1a2 haplogroup (**Supplementary Table S3**). Next, we tried to assign a haplogroup affiliation to the contaminants. We were able to identify 3 different contaminants, which belonged to haplogroups J1c2c2, H1a and K (the last one had been previously identified as contaminant[22]). We then looked in Phylotree version 15 for mutations that

unambiguously distinguish each of the contaminant haplogroups from the other contaminants and the endogenous N1b1a2. For those positions we count the number of reads matching the correspondent contaminant haplogroup in order to estimate the contribution of each contaminant to the overall contamination. On average, J1c2c2, H1a and K contaminants are present in 9.6, 13 and 1.9 % of the reads, respectively.

6.2- X chromosome contamination estimate

We followed a previously developed method[25] that lies on the fact that male individuals carry one X chromosome and thus only one allele at each site. Positions with reads showing more than one allele are a consequence of either errors (sequencing or mapping) or contamination from another individual. Given that contamination will only affect to sites where the sample and the contaminating individual present different alleles, it will lead to a higher mismatch rate in polymorphic sites than in adjacent sites that are less likely to be polymorphic.

Using 1000 Genomes Project Phase 1 data[26], we identified all the X chromosome SNPs with a frequency of 0.1 or higher in European population. This preliminary set was filtered in order to exclude SNPs less than 10 bases apart.

Using the reads data from the gourd´s genome, we counted the number of major and minor reads at SNP sites and adjacent sites (4 sites on each side of these SNP sites). Major reads are the reads carrying the allele most frequently seen in each site, and the rest are minor reads. We only analyzed sites with a minimum depth of 4 and a maximum depth of 10 in the gourd´s reads. Following Rasmussen et al. 2011[25], we performed test 1, test 2 and obtained an estimate of the contamination rate for both test 1 and test 2 using maximum likelihood (

Supplementary Table S4). Both tests yielded a contamination estimate of 19 %. We subsequently estimated the 95% confidence interval following this:

$$CI_{95\%} = (\hat{c} \pm z_{\frac{\alpha}{2}} S\hat{E}_{\hat{c}})$$

$$CI_{95\%} = (\hat{c}_{Lower}, \hat{c}_{Upper}) = (0.179, 0.200)$$

6.3- Y chromosome affiliation and contamination estimate

The presence of derived alleles at several phylogenetically relevant SNPs defining haplogroups G, G2, G2a, G2a2 and G2a2a suggests that the gourd´s genome belongs to haplogroup G2a2a

(**Supplementary Table S5**). This confirms the previous Y-chromosome attribution[22], as determined with the AmpFlSTR Identifiler PCR amplification Kit.

We noticed that several SNPs show ancestral alleles together with derived alleles. These ancestral alleles are probably due to contamination, so we used them to give an estimate of the contamination in the Y chromosome (**Supplementary Table S5**). We obtained a contamination of 17 %, which agrees with the estimate obtained for the X chromosome. We also estimated the 95% confidence interval with the following formulae: sqrt[(p*(1-p))/n)] where p is 0.17. The obtained 95CI is 0.063-0277, although due to the small number of observations the best nuclear contamination estimate is obviously that obtained from the X chromosome.

The Y chromosome affiliation was based on the International Society of Genetic Genealogy (2014), Y-DNA Haplogroup Tree 2014, Version: 9.01, Date: 1 January 2014, http://www.isogg.org/tree/, Date of access: 04 January 2014.

7- DNA fragmentation pattern

Ancient DNA sequences show characteristic damage patterns at the ends of the reads, derived from depurinations (preceding the sequencing reads) that subsequently involve deamination of exposed citosines and fragmentation of the DNA templates[27]. This process results in increased C to T substitutions at the 5' ends of the reads plus increased G to A substitutions at the 3' ends. Additionally, there is a characteristic enrichment of purines at the bases prior to 5' end of the reads and of pyrimidines after the 3' ends of the reads. While this complex pattern of DNA fragmentation is considered a signal of authenticity of ancient sequences, it is not known what should be expected from a sample as recent as the blood of king Louis XVI. The only comparable age in ancient genomic studies is that of the Australian aborigine hair sample[25], accessioned at the Duckworth Collection in Cambridge in 1923. The analysis of the damage patterns shows a mild increase –as compared to other, older samples such as Neandertals- of T's (2.3% in position 1) and A's (2.1% in position 1) at the 5' and 3' ends of the reads, respectively. Perhaps more significantly, they found a bias towards purines and pyrimidines before and after the ends, respectively.

In order to determine whether the gourd´s DNA showed the ancient DNA pattern, we analysed a random subsample of the aligned sequencing reads for their nucleotide composition and misincorporation patterns. From the SAM file containing the aligned read data from all sequencing approaches (~60 million), we randomly sampled 1% reads obtaining a similar amount of reads from

each chromosome (33,000-37,000 reads/each). To simplify the subsequent nucleotide substitution analysis, we removed reads that showed insertions or deletions with respect to the reference sequence. This final read set contained 886,789 reads with which the damage pattern analysis was performed using the mapDamage software[28]. A clear purine-pyridimine pattern is observed prior and after the gourd's reads, as well as a mild increase of damage at the ends of the reads (**Supplementary Figure S3**). Like in the case of the Australian aborigine, the excess of pyrimidines is mostly due to thymines.

8- SNP calling and filtering

The SNP calling pipeline consisted firstly in the removal of PCR duplicates with Picard tools (http://picard.sourceforge.ne), indel realignment and base quality recalibration, both with GATK[29,30], steps that were applied independently to each of the four files. The resulting files were then merged before applying the global indel realignment step with GATK. Finally, the unified genotyper and variant quality score recalibration steps were performed with GATK in order to obtain the VCF file with the SNP calls (

	e/d	***e'/d'***	***p*-value**	**OR**	**ML estimate**	**Standard error (jackknife)**
**Test1**	1564/24218	261/183186	$< 10^{-16}$	45	0.194	0.00531
**Test2**	338/5376	51/40432	$< 10^{-16}$	50	0.189	0.00997

Supplementary Table S5: Y chromosome affiliation and contamination. Phylogenetically relevant positions were ascertained in the gourd´s genome.

SNP name	Position in hg19	Haplogroup	Ancestral	Derived	Number of ancestral reads in the gourd´s genome	Number of derived reads in the gourd´s genome	Total number of reads in the gourd´s genome
L769	23059496	G	G	A	0	1	1
L116	14989721	G	C	G	0	1	1
L154	8614138	G	T	G	0	1	1
L204	5358991	G	C	G	No data	No data	No data
L240	3131153	G	G	T	No data	No data	No data
L269	14958218	G	T	C	No data	No data	No data
L402	15204708	G	T	G	No data	No data	No data
L519	8240725	G	C	T	1	2	3
L520	8700380	G	C	T	No data	No data	No data
L521	9448354	G	A	G	1	5	6
L522	17533325	G	A	C	0	1	1
L523	18957208	G	C	T	0	3	3
L605	18393536	G	G	C	1	1	2
L770	2863466	G	A	T	0	1	1
L836	16896148	G	G	A	0	1	1
L837	17853245	G	A	G	0	2	2
L1258	19431434	G	T	A	2	0	2
L1407	22023296	G	A	G	No data	No data	No data
M201	15027529	G	G	T	No data	No data	No data
P257	14432928	G	G	A	No data	No data	No data
PF3137	2846401	G	C	T	No data	No data	No data
PF2952	14577177	G	G	A	0	3	3

PF2956	14993358	G	A	G	0	2	2
PF2958	15086183	G	G	C	0	2	2
PF3134	15275200	G	C	G	No data	No data	No data
U12	14639427	G	A	C	1	0	1
U21	15204710	G	A	C	No data	No data	No data
U23	14423856	G	G	A	No data	No data	No data
L142.2	6753306	G2	G	A	0	1	1
L156	17174741	G2	A	T	0	1	1
P287	22072097	G2	G	T	0	1	1
L149.1	8426380	G2a	T	G	No data	No data	No data
L31	14028148	G2a	C	A	0	1	1
P15	23244026	G2a	C	T	0	1	1
L1259	15615340	G2a2	C	G	0	2	2
PF3146	5688132	G2a2a	C	T	No data	No data	No data
PF3147	7738069	G2a2a	G	A	No data	No data	No data
PF3151	9785736	G2a2a	A	G	No data	No data	No data
PF3159	14815695	G2a2a	C	G	No data	No data	No data
PF3161	15702713	G2a2a	A	C	0	2	2
PF3165	16582411	G2a2a	C	A	0	1	1
PF3166	16735582	G2a2a	T	G	0	1	1
PF3167	16791005	G2a2a	G	C	No data	No data	No data
PF3168	17572142	G2a2a	T	C	0	1	1
PF3172	18129746	G2a2a	A	C	No data	No data	No data
PF3175	18962113	G2a2a	C	T	No data	No data	No data
PF3176	21185138	G2a2a	G	C	No data	No data	No data
PF3180	21600446	G2a2a	A	T	0	1	1
PF3181	21808944	G2a2a	C	A	No data	No data	No data
PF3182	21822756	G2a2a	C	T	No data	No data	No data
PF3184	22576860	G2a2a	C	T	1	0	1
PF3185	22894488	G2a2a	C	T	No data	No data	No data
PF3186	23291704	G2a2a	T	C	1	0	1
					**Total**	39	47
					**Contamination**	17%	

Supplementary Table S6). The following databases were used at different steps of the pipeline: 1000G biallelic indels (indel realignment), dbSNP137 (base quality recalibration) and dbSNP137, 1000G OMNI and HapMap (variant quality score recalibration).

To ensure that the observed contamination would not contribute to any heterozygous position in our working datasets, we applied a stringent allele balance filter. We removed from the datasets all the heterozygous positions with an allele imbalance more skewed than 0.3-0.7 for the two possible alleles (based on the maximum estimated contamination, which is that from the mtDNA). We have modelled the probability that contamination remains after the allele imbalance filtering by using a Bernoulli model. We took as example sites with 12x coverage. In this case, only three allelic combinations will remain after the 30% removal, accounting for 4:8, 5:7 and 6:6 (meaning number of reads with different alleles). Using conditional probabilities and nuclear contamination estimates between 0.18 and 0.20, the remaining contamination would be (for instance, in the case of 0.18): 0.18^4 in the 4:8 combination, 0.18^5 in 5:7 and 0.18^6 in 6:6 (assuming always that the background contamination will

emerge as the minoritary allele). Thus in those SNPs of 12x coverage, the expected remaining contamination will be only between 0.25% and 0.39%.

However, this strict filtering would remove not only contaminants but many real heterozygous positions when applied to a low-coverage genome. Assuming a binomial distribution of reads with *p=0.5* for the two possible alleles, we estimated the percentage of real heterozygous variants that have been affected by the filtering as a function of coverage (**Supplementary Figure S4**). For the datasets ≥3x (mean coverage = 5), exome ≥6x (mean coverage = 9) and exome ≥9x (mean coverage = 12), we estimate that around 37.5, 28.91 and 14.60% of real heterozygous positions were lost due to the allele balance filter respectively.

9- Patterns of post-mortem damage

The predominant *post-mortem* damage patterns seen in ancient DNA sequences are C→T and G→A miscoding lesions primarily derived from the deamination of cytosines to uracils, either in the sequenced strand (C→T) or in the complementary strand, thus resulting in G→A changes[27,31]. Therefore, the number of C→T and G→A changes clearly exceeds the T→C and A→G substitutions in ancient genomes. This pattern can be observed in the exome, where the coverage is higher (**Supplementary Table S7**). For instance, taking into account all exomic reads, the frequency of C→T is 17.24% and G→A is 17.39%. In contrast, the nucleotide change frequencies of T→C and A→G are 12.02% and 12.19%, respectively.

10- PCR genotyping

A total of 9 autosomal SNPs were genotyped with a PCR approach using a two-steps protocol[32]. Sequences of primers used for amplifying each one of the fragments targeted are reported in (**Supplementary Table S8**). The first PCR step was a multiplex amplification that included all the 9 fragments with the following conditions: 2 units TaqGold DNA polymerase (Applied Biosystems), PCR buffer (1x), 4 mM MgCl2, 0.5 mM for each dNTP and 0.15 μM of each primer in a final volume of 20 μl. The second PCR step was a single amplification for each fragment and conditions were as described for the first step except that the primer concentration was increased to 1 μM. Thermocycling profiles were 12 min activation step at 94°C, followed by 27 cycles in step1 and 30 in step2 at 94°C for

20 s, 50ºC for 30 s, and 72°C for 30 s.

PCR products were visualized in a 2% low-melting point agarose gel, excised from the gel, purified with a gene clean silica method (DNA Extration Kit, Fermentas) and posteriorly cloned with the TOPO TA Cloning kit (Invitrogen), following the manufacturer's instructions. About 30 colonies for each fragment were subjected to PCR with M13 universal primers; inserts obtained were sequenced with an Applied BioSystems DNA sequencer at the Servei de Seqüenciació of the Universitat Pompeu Fabra (Barcelona).

Results confirmed the genotypes of three SNPs (**Supplementary Table S8**), determined to be heterozygous in the previous sequencing. Of the remaining six SNPs (coverage up to 6), four turned out to be heterozygous and two were homozygous, thus proving that the loss of heterozygosity is attributable to the existing low coverage.

11- Ancestry analysis

11.1- Principal component analysis

Louis XVI had a complex ancestry, with people coming from different regions of Europe, including present-day Germany, Poland, Austria, Italy and France (

Supplementary Table S9).

We first performed a principal component analysis (PCA) of gourd's SNP data at >9x coverage, using data from European individuals of the 1,000 Genomes Project for comparison. From the 236 European individuals sequenced exclusively with Illumina, only SNPs with a MAF >0.5% were considered and sites were pruned using PLINK software[33]. The LD-based SNP pruning option was set to default parameters (pairwise genotypic r2 > 0.5 within sliding windows of 50 SNPs and a overlap of 5 SNPs per sliding window) in order to retain only informative positions and avoid the possible linkage biases, producing a final set of 16,635 SNPs. Furthermore, we used 10 outlier interactions in EIGENSOFT with an outlier sigma threshold of 6.0 within the first six eigenvectors to identify possible outliers. Two individuals (HG00119 and HG00271) were removed after the first iteration on the first and third eigenvectors. Finally the PCA (**Figure 3**) was ploted using Rplot[34].

A second PCA was performed with the autosomal subset with greater than 3x coverage of the whole genome sequencing dataset. This SNP dataset was merged with a previously curated[35] POPRES dataset consisting of 1,387 unrelated European individuals for the principal components analysis (PCA). A total of 19,520 A/T or C/G SNPs were filtered from the analysis as their strandedness across

datasets are difficult to determine, leaving 101,107 SNPs shared between the gourd's genome and the POPRES dataset for PCA. PCA was performed using EIGENSTRAT version 3.0[36] with LD correction based on the regression of 2 previous SNPs, no outlier removals, and otherwise default settings. The resulting PCA plot recapitulated the general structure of Europe as expected, and placed gourd´s genome among Northern Italian and Central European samples (**Figure 4a**). Additions of highly confident genotypes from the gourd's exome sequencing did not change the PCA significantly (data not shown). PCA using exome genotypes alone produced roughly similar results, but with significantly lower resolution in delineating the population structure in Europe (data not shown).

In order to estimate the position that king Louis XVI would show in the PCA analysis, we randomly generated 30 composite genomes with the proportions of Louis XVI known ancestry, to the level of great-great grandparents. These ancestry proportions are 50 % German ancestry, 25 % Polish ancestry, 12.5 % French ancestry, 6.25 % Austrian ancestry and 6.25 % North Italian ancestry. Genotypes were selected from the curated POPRES dataset, comprising 1,387 and x SNPs. Each SNP position in the dataset was assigned to one of the 5 possible source populations, in such a way that the proportion of SNPs assigned to a particular source population is the same as the ancestry proportion of that population. Then, for each SNP a genotype was randomly picked among the samples that belonged to the corresponding source population. This procedure was repeated 30 times in order to have 30 randomly generated composite genomes. Finally, PCA was performed on the merged dataset POPRES+ composites, using EIGENSTRAT version 3.0[36] (**Figure 4b**).

11.2- Analysis of tracts of identity-by-descent

To obtain a profile of the sharing of long identity-by-descent (IBD) tracts between gourd´s individual and other individuals found in the POPRES dataset, we used the fastIBD supplied by the Beagle software. First, based on the merged dataset of gourd´s genome and POPRES, we computed for each SNP a genetic map position in centiMorgan units based on the recombination map published by deCODE[37]. We used the sex-averaged version of the map at 10kb resolution, removed any SNPs that are not within the range of the deCODE map (such as within 5 Mb of the ends of chromosomes), and linearly interpolated the genetic map position for each marker using its physical position. Additionally, we removed SNPs with a minor allele frequency less than 5% in the POPRES dataset. In total, 72,913 autosomal SNPs were used to infer IBD tracts using Beagle. We followed the recommendation of Beagle's authors[38] to merge inferred IBD tracts among the 10 independent runs, as well as a post-processing modification described previously[39], which is aimed to ameliorate Beagle's tendency to

spuriously introduce gaps into long blocks of IBD. In more detail, we first removed segments not overlapping another segment seen in at least one other run. We then merged any two segments of the same pairs of individual separated by a gap shorter than at least one of the segments and no more than 5cM long. We then discarded any segments that did not have any subsegment meeting the significance threshold of $1x10^{-8}$.

To avoid apparent differences in average sharing due to varying sample size, we down-sampled each population to a common size of 40 individuals for each POPRES population with at least 40 individuals. The populations used in the analysis include: Italy, United Kingdom, Spain, Portugal, Swiss-French, France, Swiss-German, Germany, Ireland, Belgium, and Yugoslavia. Overall, we detected six IBD tracts shared between gourd´s individual and one other POPRES individual, three of which were shared with an individual from France (**Supplementary Table S10**).

12- Functional effect characterization analysis

We restrictred the functional effect characterzation analysis to the sites that had a minimum coverage of 6 reads per site and passed the allele balance filter (section SNP calling and filtering).

Ensembl's Variant Effect Predictor v.2.5 (Ensembl 67 annotation) was used to annotate the fuctional effect of the genetic changes in the gourd´s genome. Analyses were limited to changes within the longest transcripts of CCDS-verified genes (Consensus Coding Sequence Project of EBI, NCBI, WTSI, and UCSC - Sep. 7th 2011 release). The main functional characterization of both dbSNP-137 shared and private (not shared with dbSNP-137 database) changes are summarized in **Supplementary Table S11.** Furthermore, Polyphen-2 software[40] (standalone HumDiv model) was used in order to predict the effect of non-synonymous amino acid changes. Nonsense and missense positions were analyzed in to detail to look for changes that could have a functional consequence in the gourd´s individual phenotype or fitness. We looked either for mutations occurring within genes associated to known Human Mendelian Diseases[41] or sites for which gourd´s genome is homozygote for the alternative allele (**Supplementary Table S12**

Supplementary Table S15).

For the non-synonymous changes we followed a series of different steps to ensure that we only retained the "most deleterious" changes (those that according to Polyphen-2 had a higher than 0.95 probability of being damaging and false positive rate of less than 0.05). Then we filtered the sites using a purely chemical classification, the Grantham Scores (GS)[42], and confined our analysis to sites

that had a radical Grantham Score (>= 150). Finally, we retained only positions that had a higher >4 GERP score that is correlated to the evolutionary constrains on that site[43], and hence it will be expected that changes in this positions will be deleterious (**Supplementary Table S14**

Supplementary Table S15).

13- Eye color phenotype

We checked in the gourd´s genome the state of the six most informative SNPs for eye color prediction, and used the IrisPlex[44] model to compute probabilities associated to brown, intermediate and blue eye color (**Supplementary Table S16**). We obtained probabilities of 0.892, 0.083 and 0.024 for having brown, intermediate and blue eyes, respectively.

14- Genetic risk of gourd´s individual

14.1- Empirical risk assignment

The "GWAS Catalog" is an online catalog of published genome-wide association studies that is maintained by the NHGRI[45]. The Catalog ascertains all studies that test >100,000 SNPs to compile reported SNP-trait associations that achieve $P<10^{-5}$. We used the Catalog to gain insight on the personal genetic risk of gourd´s individual for three complex phenotypes of interest: height, obesity and type 2 diabetes. Specifically, we aimed to combine information from all known associated loci to assess the fitting of the observed genetic risk of gourd´s individual to the expected distribution of risk scores in individuals of European ancestry. In the case of height, we worked with EAF (Effect Allele Frequency), the alleles that contribute to increased height, instead of risk alleles.

We selected all GWAS associations for which the associated allele was available at the GWAS Catalog (n = 7,029; accessed: 2013 March 3rd). From these, we ascertained 3,181 associations to different phenotypes for which the corresponding genotype in the gourd´s genome was available. Next, we selected all SNP-trait associations that corresponded to our phenotypes of interest, and recorded the allele frequency of the risk allele (RAF) or EAF allele gathered from HapMap (CEU individuals of European ancestry).

To calculate the expected distribution of RAF or EAF alleles in Europeans for each phenotype, we simulated 100,000 individuals with an assigned genotype at each associated SNP (we considered

Hardy-Weinberg equilibrium to draw genotypes from RAF and EAF values). For each simulated genotype, we assigned a risk score of 0 if the genotype appears to be homozygous for the protective allele, 1 if heterozygous and 2 if homozygous for the risk allele. In the case of height, the allelic load assigned was 0, 1 or 2 depending if the individual carries none, one copy or two copies of the EAF allele, respectively. Hence, the unweighted distribution of genetic risk scores in general Europeans depends on the number of associated SNPs and its RAF or EAF allele frequencies. We then used gourd´s genotypes to calculate individual RAF or EAF allele loads for each phenotype. Finally, we also constructed a weighted genetic score by means of a similar analysis, but weighting each SNP proportionally to the reported estimates of RAF or EAF effect size.

14.2- Risk allele recapturing via pairwise haplotype

Due to the low-coverage of the gourd´s genome, not all the RAF pr EAF alleles present in the NHGRI GWAS Catalog[45] associated with the phenotypes of interest could be ascertained from the genotypes in our individual. To infer the genotypes at these missing positions, we built pairwise haplotypes combining the sequenced SNPs and others in high LD, being the latter associated with the phenotype of interest. As building haplotypes from the nearly 25 million positions sequenced would be computationally expensive and time consuming, we reduced this dataset by just analyzing the genotypes resulting from crossing the gourd´s known genotypes with those present in the HapMap CEU population (because all of the HapMap SNPs have a minimum allele frequency high enough to be detected in a GWAS). This resulted in a dataset of 2,031,376 SNPs to be checked for new associations. For each of this ~ 2 million SNPs, we defined a 100 kb region centered in the SNP, with a LD threshold of $R^2 = 1$. All the SNPs fulfilling these criteria were considered and checked against the NHGRI GWAS Catalog for the phenotypes considered. Afterwards, using the 1000 Genomes data, pairwise haplotypes were built through PLINK-1.07[33], using both types of SNPs. Therefore, we could infer if the RAF or EAF allele of the non-genotyped SNP is traveling in LD with any of the LD-retrieved positions. The percentage of SNP recapturing was 35% for height, and 51% for both obesity and type 2 diabetes (**Table 1**). The latter phenotypes did not present SNPs after filtering (we considered only those SNPs recaptured by at least three neighbouring SNPs) and thus were excluded from further analyses.

14.3- Quality control

The fact that the same unknown position could be recaptured by several known SNPs, suggests that the congruency in the haplotype combination could serve as a quality control method, provided that the coverage of the genome is low and that there is a background contamination. Obviously, the probability that damage or contamination could alter a whole genomic region is lower than for single SNP positions. If the same allele for the unknown SNP is found in all or in most of the pairwise haplotypes that we retrieve from the known SNPs, we can be more confident that the specified allele is the endogenous one. Obviously, an incongruence does not necessarily mean there is an error in the genotypes, and may simply respond to the fact that the LD patterns in CEU are different from those of 250 years ago. An example of this quality control can be seen at

Supplementary Table S17.

14.4- LD patterns

To check whether we were analysing consistent and real haplotypes in the gourd's genome, we checked if haplotypes present in 60 CEU individuals from the 1000 Genomes Project Pilot phase[46] were to be found in the gourd's genome.

First, we randomly selected 244,610 SNPs from the gourd's genome and mapped those positions to the CEU genomes. Whenever mapping was possible, because the variant was called both in the gourd and in CEU, we defined a 1Mb block around that site and obtained all the SNPs in LD in CEU, using $R^2 \geq 0.7$ with PLINK software[33]. Then, those gathered positions in LD were searched, if existing, in the gourd's genome and their genotype was retrieved. We only considered homozygous positions to build the blocks, given that the gourd's genome has not been phased. With these common SNPs we retrieved all extant haplotypes and its frequency in CEU using PLINK option *--hap-freq*.

Out of these ~250 k randomly selected positions we found 17.898 haplotypes in common between CEU and the gourd's genome that were composed of the same linked variants, ranging in length from 3 to 851 SNPs. In particular, we found 10.257 haplotypes with lengths comprised between 3 and 15 SNPs, 7.262 with lengths between 16 and 100, and 379 longer than 100 SNPs. For each of the haplotypes, we compared the gourd's haplotype with the observed haplotypes in CEU, and obtained a percentage of sequence similarity. On each comparison, we took into account only the similarity value of the most similar CEU haplotype (see example in **Supplementary Table S18),** and plotted the similarity percentage for the different haplotype length bins (**Supplementary Figure S5**). For the three categories, the average similarity was > 93%, and a significant positive correlation was found between similarity to the gourd's haplotype and frequency in CEU (**Supplementary Table S19**).

As a proof of principle to validate this approach, we followed the same previous procedure using Yoruban (YRI) population. As expected, haplotype length for YRI was lower, not exceeding 253 SNPs.

We crossed both sets (CEU x YRI) to find positions where a common haplotype (not necessarily of the same length) could be built around the same SNP (n=4329). According to our expectation, those haplotypes with the highest similarity in CEU and thus more frequent, should be less frequent in YRI population or, in other words, those most frequent in YRI should have less similarity to the observed haplotype in the gourd's genome.

We found that CEU haplotypes were more similar to gourd's haplotype than YRI haplotypes when considering high frequency haplotypes. (CEU: 84% similarity; YRI: 73% similarity. Student's T-test p-value: < 0.001) (Supplementary Figure S6).

Supplementary References

1. Soderhjelm, A. *Marie-Antoinette et Barnave. Correspondance secrète.* (A. Colin, Paris, 1934).

2. Androutsos, G. Le phimosis de Louis XVI (1754-1793) aurait-il été à l'origine de ses difficultés sexuelles et de sa fécondité retardée? *Progrès en Urol.* **12,** 132–137 (2002).

3. Arneth, A. & Geffroy, M. *Marie-Antoinette. Correspondance secrète entre Marie-Thérèse et le Comte de Mercy-Argenteau.* (Firmin-Didot, Paris, 1874).

4. Besenval, P. *Mémoires De M. Le Baron De Besenval*. (F. Buisson, Paris, 1805).

5. Campan, J. *Mémoires sur la vie privée de Marie-Antoinette, reine de France et de Navarre. Suivis de souvenirs et anecdotes historiques sur les règnes de Louis XIV, de Louis XV et de Louis XVI*. (Baudouin, Paris, 1823).

6. De Lamballe, M. *Memoirs. New first published from the journal, letters, and conversations of the princess Lamballe by a Lady of Rank. With a portrait and cipher of the secret correspondence of Marie-Antoinette.* (Treuttel and Würtz, London, 1826).

7. De Lescure, M. *Correspondance secrete inédite sur Louis XVI, Marie-Antoinette, la Cour et la ville de 1777 à 1792*. (Henri Plon, Paris, 1866).

8. Fogg, R. & Boorjian, S. The sexual dysfunction of Louis XVI: a consequence of international politics, anatomy, or naïveté? *BJU Int.* **106,** 457–459 (2010).

9. Ganière, P. Louis XVI a-t-il eu recours à la lancette? *Presse Med.* **72,** 3401–3406 (1964).

10. Girault de Coursac, P. *Louis XVI, un visage retrouvé : portrait physique et moral du dernier roi très Chrétien.* (OEIL, Paris, 1990).

11. Levier, E. *Marie-Antoinette*. (Fayard, 1991).

12. Nicolardot, L. *Journal de Louis XVI*. (E. Dentu, Paris, 1873).

13. Nougaret, P. *Anecdotes du règne de Louis XVI*. (1791).

14. Soulavie, A. *Mémoires historiques et politiques du règne de Louis XVI.* (Treuttel and Würtz, Paris, 1801).

15. Lalueza-Fox, C. *et al.* A melanocortin 1 receptor allele suggests varying pigmentation among Neanderthals. *Science* **318,** 1453–1455 (2007).

16. Marco-Sola, S., Sammeth, M., Guigó, R. & Ribeca, P. the Gem mapper : fast , accurate and versatile alignment by filtration. *Nat. Methods* **9,** (2012).

17. Minoche, A. E., Dohm, J. C. & Himmelbauer, H. Evaluation of genomic high-throughput sequencing data generated on Illumina HiSeq and genome analyzer systems. *Genome Biol.* **12,** R112 (2011).

18. Zerbino, D. R. & Birney, E. Velvet: algorithms for de novo short read assembly using de Bruijn graphs. *Genome Res.* **18,** 821–9 (2008).

19. Huson, D. H., Auch, A. F., Qi, J. & Schuster, S. C. MEGAN analysis of metagenomic data. *Genome Res.* **17,** 377–86 (2007).

20. Lindgreen, S. AdapterRemoval: easy cleaning of next-generation sequencing reads. *BMC Res. Notes* **5,** 337 (2012).

21. Li, H. & Durbin, R. Fast and accurate short read alignment with Burrows–Wheeler transform. *Bioinformatics* **25,** 1754–1760 (2009).

22. Lalueza-Fox, C. *et al.* Genetic analysis of the presumptive blood from Louis XVI, King of France. *Forensic Sci. Int. Genet.* **5,** 459–63 (2011).

23. Kloss-Brandstätter, A. *et al.* HaploGrep: a fast and reliable algorithm for automatic classification of mitochondrial DNA haplogroups. *Hum. Mutat.* **32,** 25–32 (2011).

24. Van Oven, M. & Kayser, M. Updated comprehensive phylogenetic tree of global human mitochondrial DNA variation. *Hum. Mutat.* **30,** E386–94 (2009).

25. Rasmussen, M. *et al.* An Aboriginal Australian genome reveals separate human dispersals into Asia. *Science* **334,** 94–8 (2011).

26. Consortium, 1000 Genomes Project. An integrated map of genetic variation from 1,092 human genomes. *Nature* **491,** 56–65 (2012).

27. Briggs, A. W. *et al.* Patterns of damage in genomic DNA sequences from a Neandertal. *Proc. Natl. Acad. Sci. U. S. A.* **104,** 14616–21 (2007).

28. Ginolhac, A., Rasmussen, M., Gilbert, M. T. P., Willerslev, E. & Orlando, L. mapDamage: testing for damage patterns in ancient DNA sequences. *Bioinformatics* **27,** 2153–5 (2011).

29. McKenna, A. *et al.* The Genome Analysis Toolkit: a MapReduce framework for analyzing next-generation DNA sequencing data. *Genome Res.* **20,** 1297–303 (2010).

30. DePristo, M. a *et al.* A framework for variation discovery and genotyping using next-generation DNA sequencing data. *Nat. Genet.* **43,** 491–8 (2011).

31. Hofreiter, M., Jaenicke, V., Serre, D., von Haeseler, a & Pääbo, S. DNA sequences from multiple amplifications reveal artifacts induced by cytosine deamination in ancient DNA. *Nucleic Acids Res.* **29,** 4793–9 (2001).

32. Krause, J. *et al.* The derived FOXP2 variant of modern humans was shared with Neandertals. *Curr. Biol.* **17,** 1908–12 (2007).

33. Purcell, S. *et al.* PLINK : A Tool Set for Whole-Genome Association and Population-Based Linkage Analyses. *Am. J. Hum. Genet.* **81,** 559–575 (2007).

34. R Core Team. R: A Language and Environment for Statistical Computing. R Foundation for Statistical Computing, Vienna, Austria. (2012).

35. Novembre, J. *et al.* Genes mirror geography within Europe. *Nature* **456,** 98–101 (2008).

36. Price, A. L. *et al.* Principal components analysis corrects for stratification in genome-wide association studies. *Nat. Genet.* **38,** 904–9 (2006).

37. Kong, A. *et al.* Fine-scale recombination rate differences between sexes, populations and individuals. *Nature* **467,** 1099–1103 (2010).

38. Browning, B. L. & Browning, S. R. A fast, powerful method for detecting identity by descent. *Am. J. Hum. Genet.* **88,** 173–82 (2011).

39. Ralph, P. & Coop, G. The geography of recent genetic ancestry across Europe. *PLoS Biol.* **11,** e1001555 (2012).

40. Adzhubei, I. a *et al.* A method and server for predicting damaging missense mutations. *Nat. Methods* **7,** 248–9 (2010).

41. Blekhman, R. *et al.* Natural selection on genes that underlie human disease susceptibility. *Curr. Biol.* **18,** 883–9 (2008).

42. Grantham, R. Amino acid difference formula to help explain protein evolution. *Science* **185,** 862–864 (1974).

43. Davydov, E. V *et al.* Identifying a high fraction of the human genome to be under selective constraint using GERP++. *PLoS Comput. Biol.* **6,** e1001025 (2010).

44. Walsh, S. *et al.* IrisPlex: a sensitive DNA tool for accurate prediction of blue and brown eye colour in the absence of ancestry information. *Forensic Sci. Int. Genet.* **5,** 170–80 (2011).

45. Hindorff, L. a *et al.* Potential etiologic and functional implications of genome-wide association loci for human diseases and traits. *Proc. Natl. Acad. Sci. U. S. A.* **106,** 9362–7 (2009).

46. Abecasis, G. R. *et al.* A map of human genome variation from population-scale sequencing. *Nature* **467,** 1061–73 (2010).
